# METTL14 aggravates endothelial inflammation and atherosclerosis by increasing FOXO1 N6-methyladeosine modifications

**DOI:** 10.7150/thno.45178

**Published:** 2020-07-11

**Authors:** Dongdong Jian, Ying Wang, Liguo Jian, Hao Tang, Lixin Rao, Ke Chen, Zhen Jia, Wanjun Zhang, Yiran Liu, Xu Chen, Xiwen Shen, Chuanyu Gao, Shuai Wang, Muwei Li

**Affiliations:** 1 Department of Cardiology, Henan Provincial People's Hospital, Department of Cardiology of Central China Fuwai Hospital, Henan Key Laboratory for Coronary Heart Disease Prevention and Control, Central China Fuwai Hospital of Zhengzhou University, Zhengzhou, Henan, 450003, China.; 2 Department of Cardiology, The Second Affiliated Hospital of Zhengzhou University, Zhengzhou, Henan, 450003, China.; 3 Heart Center of Henan Provincial People's Hospital, Central China Fuwai Hospital, Key Laboratory for Cardiac Regenerative Medicine, National Health Commission & Central China Fuwai Hospital of Zhengzhou University, Zhengzhou, Henan, 450003, China.; 4 Department of Cardio-Thoracic Surgery, The First Affiliated Hospital, School of Medicine, Zhejiang University, Hangzhou, Zhejiang, 310003, China.; 5 Department of Hematology, Henan Provincial People's Hospital, Zhengzhou, Henan, 450003, China.; 6 Department of Pathology, Henan Provincial People's Hospital, Zhengzhou, Henan, 450003, China.; 7 Department of Hepatobiliary and Pancreatic Surgery, Zhengzhou People's Hospital, Zhengzhou, Henan, 450003, China.; 8 School of Life Sciences, Westlake University, Hangzhou, Zhejiang, 310024, China.

**Keywords:** METTL14, m^6^A modification, endothelial inflammation, FOXO1, atherosclerosis

## Abstract

**Aims:** The N6-methyladenosine (m^6^A) modification plays an important role in various biological processes, but its role in atherosclerosis remains unknown. The aim of this study was to investigate the role and mechanism of m^6^A modification in endothelial cell inflammation and its influence on atherosclerosis development.

**Methods:** We constructed a stable TNF-α-induced endothelial cell inflammation model and assessed the changes in the expression of m^6^A modification-related proteins to identify the major factors involved in this process. The m^6^A-modified mRNAs were identified by methylated RNA immunoprecipitation (RIP) sequencing and forkhead box O1 (FOXO1) was selected as a potential target. Through cytological experiments, we verified whether methyltransferase-like 14 (METTL14) regulates FOXO1 expression by regulating m^6^A-dependent mRNA and protein interaction. The effect of METTL14 on atherosclerosis development *in vivo* was verified using METTL14 knockout mice.

**Results:** These findings confirmed that METTL14 plays major roles in TNF-α-induced endothelial cell inflammation. During endothelial inflammation, m^6^A modification of FOXO1, an important transcription factor, was remarkably increased. Moreover, METTL14 knockdown significantly decreased TNF-α-induced FOXO1 expression. RIP assay confirmed that METTL14 directly binds to FOXO1 mRNA, increases its m^6^A modification, and enhances its translation through subsequent YTH N6-methyladenosine RNA binding protein 1 recognition. Furthermore, METTL14 was shown to interact with FOXO1 and act directly on the promoter regions of *VCAM-1* and *ICAM-1* to promote their transcription, thus mediating endothelial cell inflammatory response. *In vivo* experiments showed that METTL14 gene knockout significantly reduced the development of atherosclerotic plaques.

**Conclusion:** METTL14 promotes FOXO1 expression by enhancing its m^6^A modification and inducing endothelial cell inflammatory response as well as atherosclerotic plaque formation. Decreased expression of METTL14 can inhibit endothelial inflammation and atherosclerosis development. Therefore, METTL14 may serve as a potential target for the clinical treatment of atherosclerosis.

## Introduction

Endothelial inflammatory response is usually caused by various inflammatory factors and results in increased endothelial cell permeability, impaired barrier function, and expression of adhesion molecules, such as intercellular adhesion molecule 1 (ICAM-1), vascular cell adhesion molecule 1 (VCAM-1), and E-selectin. This leads to increased blood monocyte adhesion and migration into the sub-endothelial space, which in turn triggers the occurrence and development of atherosclerosis 1-3. The persistence of this situation eventually leads to diseases such as cardiovascular diseases, including atherosclerosis, ischemia/reperfusion injury, and allograft rejection 4. An important event in the endothelial inflammatory response is the localized and restricted recruitment of blood leukocyte subsets to the vessels through endothelial-dependent mechanisms. In the process of endothelial-mononuclear adhesion, VCAM-1, ICAM-1, E-selectin, and other adhesion molecules play important roles as bridge connections mediating the adhesion of monocytes to endothelial cells in the blood circulation and their migration into the intima 5, 6. Therefore, exploring new pathways that regulate the expression of adhesion molecules may result in organized mononuclear-endothelial adhesion from the source and inhibit the development of atherosclerosis.

N6-methyladenine (m^6^A) modification, a type of epigenetic modification, is the most common and abundant RNA molecular modification in eukaryotes, accounting for 80% of RNA base methylation modifications 7. In transcribed mRNA, m^6^A modification can affect the processing and metabolism of mRNA, including the secondary structure, subcellular localization, alternative polyadenylation, nuclear transport, translation ability, and degradation 8, 9. The m^6^A methylation modification of RNA is mediated by a polyprotein complex and comprises three steps: writing, mainly executed by methyltransferase-like 3 (METTL3), METTL14, and WTAP (WT1 associated protein) 10; erasing, mainly executed by demethylase FTO (alpha-ketoglutarate-dependent dioxygenase FTO) and AlkB homolog 5, RNA demethylase 11, 12; and recognizing, where YTH N6-methyladenosine RNA binding protein 1 (YTHDF1) and YTHDF3 recognize m^6^A-modified RNA and promote mRNA translation. Eukaryotic translation initiation factor 3 subunit A directly binds to the m^6^A site at the 5'-Untranslated region (UTR) of the mRNA to participate in translation initiation. YTHDF2 binds to the m^6^A modification site, which leads to mRNA degradation 13, 14. Recently published studies have shown that m^6^A modification occurs in most species and plays an important role in many biological processes, including development, metabolism, and reproduction 15. However, studies on the role of m^6^A modifications in atherosclerosis are limited and the underlying mechanism remains to be elucidated in detail.

In this study, we found that METTL14 plays an important regulatory role in endothelial cell inflammation. By regulating the m^6^A modification of forkhead box O1 (FOXO1) mRNA, METTL14 promotes the translation of FOXO1 mRNA through YTHDF1 recognition, thereby upregulating the expression of the endothelial adhesion molecules VCAM-1 and ICAM-1, which promotes mononuclear-endothelial adhesion, ultimately leading to the development of atherosclerosis. Our study not only demonstrates, for the first time, the role of m^6^A modifications in the development of endothelial cell inflammation and atherosclerosis, but also highlights a potential target for the prevention and treatment of atherosclerosis.

## Methods

### Materials

An antibody against METTL14 was purchased from Novus Biological (Cat.No.NBP1-81392, Novus Biological, Colorado, USA). An antibody against m^6^A was purchased from Synaptic Systems (Cat. No. 202003, Synaptic Systems, Goettingen, Germany). Antibodies against METTL3 (Cat.No.ab195352), WTAP (Cat. No. ab155655), GAPDH (Cat. No. ab181602), CD31 (Cat. No. ab28364), FOXO1 (Cat. No. ab52857), YTHDF1 (Cat. No. ab220162), YTHDF2 (Cat. No. ab220163), YTHDF3 (Cat. No. ab220161), KIAA1429 (Cat. No. ab178966), FTO (Cat. No. ab124892), ALKBH5 (Cat. No. ab69325), VCAM-1 (Cat. No. ab134047), and ICAM-1 (Cat. No. ab2213) were purchased from Abcam (Abcam, USA). Antibodies against Flag (Cat. No. F7425), Myc (Cat. No. A7470) were purchased from Sigma-Aldrich (Sigma, USA). An antibody against E-selectin was purchased from Santa Cruz (Cat. No. sc-71017, Santa Cruz, CA). TNF-α was purchased from R&D Systems (Cat. No. 210-TA, R&D Systems, USA) and dissolved in phosphate buffered saline (PBS) to a final concentration of 10 μM.

### Animals

METTL14 heterozygous mice (Mettl14^+/-^) were generated on the C57/BL6 background by Cyagen Biosciences (Suzhou, Jiangsu, China) using CRISPR/Cas9-based targeting and homology-directed repair. The targeted region of the Mettle14 gene were: gRNA1 (matching the forward strand of gene): TGCCTGTGTATAGTAACGTCAGG; gRNA2 (matching the reverse strand of gene): ATTATTACTGGGTGGTGTACAGG. In founder lines, the lack of mutations in off-target genes was verified by PCR amplification and sequencing of select candidate genes. METTL14^+/-^ mice are generated by mating wild-type mice (C57/BL6 background) with METTL14^+/-^ mice. METTL14^+/-^/APOE^-/-^ healthy offspring mice are produced by heterozygous METTL14^+/-^ mice and heterozygous APOE^-/-^ mice by Mendelian ratios. APOE^-/-^ mice and C57/BL6 mice were purchased from Model Animal Research Center of Nanjing (Nanjing, Jiangsu, China). All mice were housed in the Laboratory Animals Center of the Henan Provincial People's Hospital, with controlled temperature and humidity and a 12:12-hour dark-light cycle, and were provided water and mouse chow ad libitum. All experimental procedures were approved by the Animal Care Ethics Committee of the Zhengzhou University and Henan Provincial People's Hospital, and were performed in accordance with the American Physiology Society's “Guides for the Care and Use of Laboratory Animals” published by the National Institutes of Health.

### Assessment of Atherosclerotic Lesions in the Aorta and Aortic Sinus

APOE-/- and METTL14+/-/ APOE-/- mice were fed with a high fat high cholesterol diet (Western Diet) for 12 weeks. The mice were anesthetized with sodium pentobarbital (50 mg/kg, i.p.; BHD, Toronto, Ontario, Canada). After exposing the abdominal cavity, the sternum was opened and the thoracic cavity was exposed. After rapid opening of the right atrium, 5 ml of saline was slowly injected into the left ventricle along the apex of the heart to wash the blood. Next, 4% polyformaldehyde was slowly injected to fix the vascular tissue morphology for 30 min. Thereafter, the aorta and perivascular adipose tissue were removed under a stereomicroscope, the pulmonary arteriovenous malformation was excised, and then the artery together with the heart was removed, followed by placement in 4% paraformaldehyde at room temperature overnight. The aortas were dissected, and whole aortas were opened longitudinally from the aortic arch to the iliac bifurcation, mounted en face, and stained for lipids with Oil Red O (Nanjing Jiancheng Bioengineering Institute, Nanjing, Jiangsu, China). The hearts were embedded in OCT (Tissue-Tek; Sakura, Torrance, CA), and serial 5-mm-thick cryosections from the aortic sinus were mounted and stained with Oil Red O and hematoxylin-eosin. Image analysis was performed by a trained observer blinded to the genotype of the mice. Representative images were obtained, and lesion areas were quantified with Image J. The lesion area in the aorta en face preparations was expressed as a percent of the aortic surface area, as previously reported 16, 17.

### Immunofluorescence and Confocal Laser Scanning Microscopy

The collected vascular tissue was embedded in paraffin and sliced at a thickness of 5 μm using a microtome. The sample was attached to a glass slide, and each segment was cut into 3-5 sheets at intervals of 50 μm. After dewaxing, the sample was permeabilized in 0.5% TritonX-100 (prepared in PBS) at room temperature for 20 min, was blocked with PBS containing 10% normal goat serum for 1 h at room temperature, and then was incubated overnight at 4 °C with the primary antibody diluted with 5% BSA (1:50-1:200). Next, the sample was washed 3 times with PBS for 10 min each, and then was incubated with fluorescently labeled secondary antibody (1:200) of the corresponding species for 1 hour at 37 °C in the dark. Thereafter, the sample was washed 3 times with PBS for 10 min each and then was incubated with the appropriate amount of DAPI in the dark for 10 min. Next, the sample was washed with PBS 3 times for 10 min each, and then anti-fluorescence quencher was added. Finally, the fluorescence signal was monitored by confocal laser scanning microscopy (Olympus, Japan).

### Cell culture

Primary human umbilical vein endothelial cells (HUVECs) were obtained from Sciencell (CA, USA) and cultured in the Endothelial Cell Medium (Sciencell, CA, USA). Cells were passed 2-6 times for this study. THP-1 was obtained from the ATCC (Manassas, VA, USA) and maintained in RPMI 1640 culture medium containing 10% FBS, 100U/ml penicillin and 100 μg/ml streptomycin (all from Invitrogen, USA). Human embryonic kidney 293T cells were obtained from the ATCC (Manassas, VA, USA) and maintained in Dulbecco's modified Eagle's medium (DMEM) containing 10% fetal bovine serum (Gibco, North America), penicillin (100 units/ml), and streptomycin (20 units/ml). All cells were cultured at 37 °C and 5% CO2 in a humidified incubator.

### Quantification of the m^6^A modification

Total RNA was isolated using TRIzol reagent (Invitrogen, USA) according to the manufacturer's instructions and then was treated with deoxyribonuclease I (Sigma-Aldrich, USA). The RNA quality was analyzed using the NanoDrop2000 system. mRNA was further purified using the GenElute Direct mRNA Miniprep kit (Merck, USA). The change in global m^6^A levels in the mRNA was measured using the EpiQuik m6A RNA Methylation Quantification Kit (Colorimetric; Epigentek, P-9005-48) following the manufacturer's protocol. Poly-A-purified RNA (200 ng) was used for each sample analysis. Briefly, 200 ng of RNA was used to coat the assay wells. The capture antibody solution and detection antibody solution were then added to the assay wells separately using a suitable diluted concentration. The m^6^A levels were colorimetrically quantified by reading the absorbance at a wavelength of 450 nm, and then calculations were performed based on the standard curve.

### Lentivirus packaging and titer determination

Lentivirus packaging and titer determination was performed as described previously 18. Briefly, full-length of human METTL14 or FOXO1 cDNA was generated by PCR reaction and constructed into a pLVX-IRES-Neo plasmid. The lentivirus shuttle plasmid and the auxiliary packaging original plasmid were synthesized. All three constructed plasmids were separately subjected to high-purity endotoxin-free extraction, and co-transfected in 293T cells. The cell supernatant containing the recombinant plasmids was collected by centrifugation after culture for 48h, and the virus-containing supernatant was concentrated by ultracentrifugation (100,000g, 2h). Then, the virus titer was determined by limited dilution method as mentioned previously 18. MOI=50 of the Lv-M14 (lentivirus-METTL14) or Lv-FOXO1 (lentivirus-FOXO1) and Lv-Null (Control lentivirus) were used in this experiment.

### siRNA transfection

METTL14 siRNA (si-M14), FOXO1 siRNA (si-FOXO1), YTHDF1 siRNA (si-YTHDF1), YTHDF2 siRNA (si-YTHDF2), YTHDF3 siRNA (si-YTHDF3) and related negative control siRNA (si-NC) were all purchased from RiboBio Co., Ltd. (Guangzhou, China). HUVECs or 293T cells were transfected with 50 nM siRNA using Lipofectamine RNAiMAX reagent (Life technologies, USA) according to the manufacturer's instructions. Cells were harvested after transfection for 48-72 hours for the following experiment.

### Western Blotting

Cells were homogenized in RIPA buffer (Beyotime, China) supplemented with protease and phosphatase inhibitors (Beyotime, China) at 4 °C for 30 min. After centrifuged at 12,000g for 10 min at 4 °C, the supernatant of lysates were collected and determined by Pierce™ BCA Protein Assay Kit (Thermo, USA) according to the user guidance. Equal solubilized proteins were run on a 10% polyacrylamide SDS gels and transferred onto polyvinylidene difluoride (PVDF) membranes (Millipore, USA). Then the membranes were incubated with 5% skimmed milk powder (BD Biosciences, USA) for 1 h at room temperature and after incubation, soaked in indicated antibodies overnight at 4 °C. The next day, blotting with a horseradish peroxidase conjugated secondary antibody (Beyotime, China) for 1 hour at room temperature and then visualizing by ECL Kit (Millipore, USA). Chemiluminescence was detected by exposure to film; quantification was performed using Image J.

### Reverse transcription-quantitative PCR (RT-qPCR)

Using a total mRNA isolation kit (Tiangen, China) to isolate total cellular mRNA according to the manufacturer's instructions. First-strand cDNA was generated from 500ng of total mRNA using the PrimeScript mRNA RT reagent kit (Takara, Japan). qPCR was performed using SYBR-Green Premix Ex Taq (Takara, Japan) according to the instructions. The ABI PRISM 7500 Sequence Detection System (Life Technologies, USA) was used to measure the fluorescence degree. Gene expression was calculated using the ^ΔΔ^CT method.

### Monocyte Adhesion

HUVECs were transfected with the relevant overexpression or control lentivirus and knockdown or negative control siRNAs for 48 h, followed by treatment with 10 ng/mL of TNF-α for 6 h. To assess the binding of THP-1 cells to HUVECs, THP-1 cells were labeled with 5-(and 6)-carboxyfluorescein diacetate and succinimidyl ester (MedChemExpress, Monmouth Junction, NJ) according to the manufacturer's protocols and then were incubated with the HUVECs for 30 min at 37 °C. Unbound cells in the dishes were removed by washing 3 times with PBS. Adherent cells were visualized under an inverted fluorescence microscope and were counted in 5 randomly selected fields in each well.

### Dot-blot assay

Dot-blot assay was performed as described previously 19. Briefly, the total RNA of HUVECs was isolated with TRIzol reagent (Invitrogen, USA). 200ng RNA per group (1.5µl) and two-fold dilution was denatured by heating at 72 °C for 5 min, followed by chilling on ice immediately, and then transferred onto a nitrocellulose membrane (Amersham, GE Healthcare, USA). The membranes were then UV cross-linked, blocked, and incubated with an m6A-specific antibody (Synaptic Systems, 202003, 1:1000); the other membrane was stained with methylene blue as a loading control.

### Chromatin Immunoprecipitations (ChIPs), Re-ChIPs and Immunoprecipitations (IPs)

ChIP was performed with modifications of the procedure described by Raphaël et al 20. Chromatin was crosslinked using 1.5% formaldehyde for 5 min at 37 °C. HUVECs were maintained in ECM at 37°C under 5% CO_2_ and were collected after twice washes with PBS. 2×10^6^ cells were diluted in 1 ml of cell collection buffer (100 mM Tris-HCl [pH 9.4] and 100 mM DTT). The cell suspension was then incubated on ice for 15 min and subsequently at 30 °C for 15 min. The cells were then lysed sequentially by vortexing and 5 min centrifugation at 3000×g at 4 °C with 1 ml of Buffer A (10 mM EDTA, 0.5 mM EGTA, 10 mM HEPES [pH 6.5] and 0.25% Triton X-100) and 1 ml of Buffer B (1 mM EDTA, 0.5 mM EGTA, 10 mM HEPES [pH 6.5] and 200 mM NaCl), followed by sonication twice per 15 s at maximum settings in 100 µl of lysis buffer [10 mM EDTA, 50 mM Tris-HCl [pH 8.0], 1% SDS, 0.5% Empigen BB (Sigma)]. After centrifugation, 15 µl of the supernatant was used as the input, and the remainder was diluted 2.5-fold in IP buffer (2 mM EDTA, 100 mM NaCl, 20 mM Tris-HCl [pH 8.1], and 0.5% Triton X-100). This diluted fraction was subjected to immunoprecipitation with the indicated antibodies overnight after 2 h of preclearing at 4 °C with 20 µl of pre-immune IgG (Sigma), 0.05% BSA, 5 µg of sheared salmon sperm DNA, and 50 µl of a 50% protein A-Sepharose beads (Sigma) slurry. These beads were prepared by washing three times in PBS and resuspended in 1 mM EDTA and 10 mM Tris-HCl [pH 8.1]. Complexes were recovered by 2 h incubation at 4 °C with 2 µg of sheared salmon sperm DNA and 50 µl of protein A-Sepharose. Precipitates were serially washed with 300 µl of Washing Buffer I (2 mM EDTA, 20 mM Tris-HCl [pH 8.0], 0.1% SDS, 1% Triton X-100, 150 mM NaCl), Washing Buffer II (2 mM EDTA, 20 mM Tris-HCl [pH 8.0], Washing Buffer III (1 mM EDTA, 10 mM Tris-HCl [pH 8.0], 1% NP-40, 1% deoxycholate, 0.25 M LiCl) and then twice with 1 mM EDTA, 10 mM Tris-HCl [pH 8.0]. the precipitated chromatin complexes were removed from the beads through a 30 min incubation with 50 µl of 1% SDS, 0.1 M NaHCO_3_, with vortexing each 5 min. This step was repeated twice, with 10 min incubation times. In Re-ChIP experiments, the complexes were eluted by incubation for 30 min at 37 °C in 25 µl of 10 mM DTT. After centrifugation, the supernatant was diluted 20 times with Re-ChIP buffer (1% Triton X-100, 2 mM EDTA, 150 mM NaCl, 20 mM Tris-HCl, [pH 8.1]) and was subjected again to the ChIP procedure. All buffers contained 1× protease inhibitor cocktail (Complete minus EDTA; Roche). Crosslinking was reversed by overnight incubation at 65 °C. DNA was purified using QIAquick columns (Qiagen, Hilden, Germany), as indicated by the manufacturer, except that the samples were first mixed with agitation for 30 min with PB buffer. For the co-immunoprecipitation, 5×10^7^ cells were lysed in RIPA buffer (1% NP-40, 0.5% sodium deoxycholate, 0.1% SDS in PBS), followed by sonication twice for 15s. The retained complexes were washed as described above. After immunoprecipitation as in the ChIP procedure, the proteins were transferred onto PVDF membranes (Millipore, Germany), and the blots were developed using the ECL kit (Millipore, Germany).

### Polysome Fractionation Assay

Polysome fractionation assay was performed as described previously 21. Briefly, HUVECs were transfected with si-YTHDF1 and treated with TNF-α. After the cells were treated with 100 mg/ml cycloheximide (Sigma, USA) for 10 min at 37 °C, 500 μl of cellular cytoplasmic extract was layered onto a 10-50% sucrose gradient and centrifuged at 39,000 rpm in a rotor (Beckman, USA) for 6 h at 4 °C. The samples from the top of the gradient into were separated into 15 fractions and analyzed by QRT-PCR.

### Plasmid construction and Dual-luciferase reporter assay

Recombinant vectors encoding the FOXO1 cDNA sequence and containing the amino acids approximately 200 bp upstream and downstream of different GGACU sites (CDS-1, CDS-2, CDS-3, and 3'-UTR) and mutation sites (GGACU site of the 3'-UTR was mutated into GGCCU) was chemically synthesized by Sangon Biotech (Shanghai, China) and then inserted into downstream of firefly luciferase in the pMIRREPORT vector (Luciferase miRNA Expression Reporter Vector, Ambion). All constructs were confirmed by DNA sequencing. Subsequently, 2×10^4^ HUVECs were seeded in 24-well plates the day before transfection. The 3'-UTR recombinant vector or mutated vector (400 ng), and the renilla luciferase plasmid (pGL-TK, an endogenous control; 50 ng) were co-transfected into HUVECs using jetPEI-HUVEC (Polyplus transfection, Strasbourg, France) according to the manufacturer's instructions. Cells were washed and lysed with reporter gene lysis buffer (Promega, USA) after transfection for 48 h. Firefly luciferase activity was detected using a luminometer with a dual-luciferase reporter assay system (Promega, USA) and normalized to control Renilla luciferase levels.

### RNA-immunoprecipitation (RIP) and MeRIP-seq

For RIP assay, cells were harvested and lysed in polysome lysis buffer [100 mM KCl, 10 mM HEPES (pH 7.0), 0.5% NP40, 5 mM MgCl_2_, 1 mM DTT, 80 U/ml RNase inhibitors, and protease inhibitor cocktail] for 10 min on ice. Cell lysates were sonicated to fragment chromatins and RNAs and centrifuged. The protein concentration in the supernatant was measured with the BCA protein assay kit (Thermo Fisher Scientific, USA). Proteins were incubated with Protein G-coupled Magnetic Dynabeads (Life Technologies, USA) pre-coated with rabbit anti-METTL14 antibody, anti-FOXO1 antibody, anti-m6A antibody, or IgG control overnight at 4°C. Prior to the incubation, 1/10 of the supernatant was set aside to be used as input. After incubation, samples were washed 5 times with NT buffer [50 mM, Tris-HCl (pH 7.4), 1 mM MgCl_2_, 150 mM NaCl, and 1% Triton X-100]. Immunoprecipitated RNAs and input RNAs were extracted using TRIzol reagent (Invitrogen). For meRIP-seq analysis, reads uniquely mapped to the genome were subjected to calling the peaks in the enriched regions with a fold enrichment of at least 2 over input reads.

### Biotin-tagged RNA Pulldown

Biotin-tagged RNA pull-down assays were performed as follows: Briefly, biotin-labeled wile type mRNA containing the m6A site in the 3'-UTR (biotin-WT) or the mutated m6A site (biotin-Mut) was constructed using an in vitro transcription and biotin RNA labeling mix (Merck, USA). Then, equal amounts of biotin-WT or biotin-Mut were incubated with HUVEC lysates and streptavidin Dynabeads at room temperature for 1 h. After the supernatant was removed, streptavidin Dynabeads were washed twice and boiled for 10 min at 100 °C in loading buffer. Finally, the samples were subjected into western blotting analysis.

### Interactome Analysis with Mass Spectrometry

For the interactome analysis, 293T cells were grown, and nuclear extracts were prepared as described 22. The protein concentration was determined with a BCA assay. Protein extracts were incubated with anti-METTL14 beads (Chromotek, München, Germany) or with blocked agarose beads (Chromotek, München, Germany) as a negative control on a rotating wheel at 4 °C with 1 mg input protein. The proteins were subjected to SDS-PAGE, and the gels were then stained with the Fast Silver Stain Kit (Beyotime, Shanghai, China). Proteins specifically interacting with METTL14 were identified by reverse-phase liquid chromatography coupled with tandem mass spectrometry (ACQUITYTM UPLC-QTOF) at Beijing Protein Innovation (Beijing, China).

### Statistical Analysis

The results are presented as means ± SEM. Statistical analysis was performed using SPSS 23.0 (SPSS Software). The Shapiro-Wilk normality test was used to check the normality of the data. The data with a Shapiro-Wilk test *P*>0.05 was considered to fit a normal distribution. Two-tailed unpaired Student's *t* test was used for comparisons between groups, and one-way ANOVA with Bonferroni's post-hoc test was applied when >2 groups were compared if the data displayed a normal distribution. α=0.05 was chosen as the significance level, and *P* value <0.05 was considered statistically significant. All experiments were performed at least three times.

## Results

### METTL14 is upregulated in TNF-α-induced endothelial inflammation and atherosclerotic lesions

Endothelial inflammatory response is the initial step in atherosclerosis. Previous studies have shown that TNF-α can induce significant endothelial inflammation, mainly characterized by increased expression of the endothelial cell adhesion molecules ICAM-1, VCAM-1, and E-selectin 23, 24. Therefore, in this study, we first constructed a stable model of TNF-α-induced endothelial cell inflammation. Previous studies have shown that 10 ng/ml of TNF-α can significantly induce endothelial inflammatory response. Thus, we stimulated HUVECs with 10 ng/ml of TNF-α for different time periods and found that the expression of adhesion molecules was significantly induced at 2 to 12 h (Figure S1A). HUVECs were then stimulated with different concentrations of TNF-α for 6 h. Consistent with previous studies, we found that 10 ng/ml of TNF-α induced significant endothelial inflammation (Figure S1B). Therefore, cells were treated with 10 ng/ml of TNF-α for 6 or 12 h to establish a model of endothelial inflammation.

**Figure 1:**
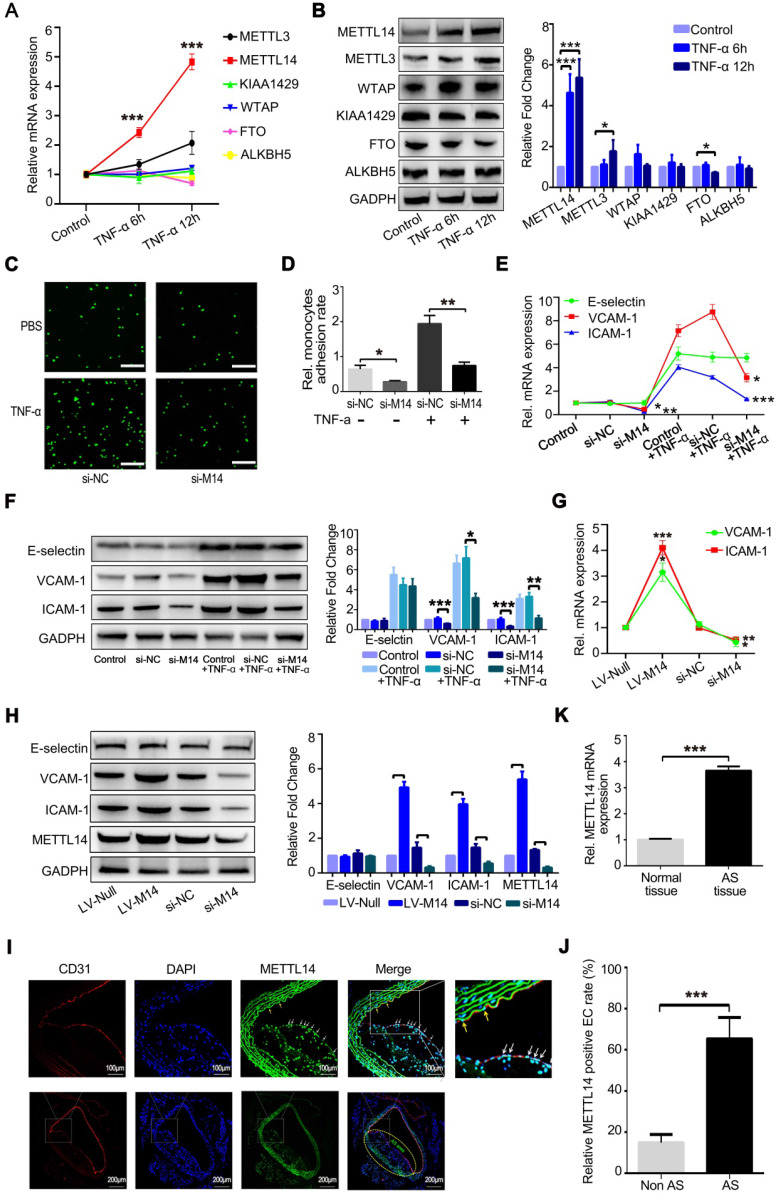
** METTL14 is increased in TNF-α-induced endothelial cells and atherosclerotic lesions.** (**A**) QRT-PCR detection of METTL3, METTL14, KIAA1429, WTAP, FTO and ALKBH5 in HUVECs stimulated with 10 ng/ml TNF-α for 6 h or 12 h. (**B**) Representative western blot results of METTL3, METTL14, KIAA1429, WTAP, FTO and ALKBH5 after TNF-α stimulation in HUVECs. (**C and D**) Endothelial mononuclear adhesion assays in HUVECs after knockdown of METTL14 (with or without TNF-α stimulation) and the statistics of the relative monocyte adhesion rate. (**E**) QRT-PCR detection of VCAM-1, ICAM-1 and E-selectin in HUVECs stimulated with 10 ng/ml TNF-α for 12 h with or without METTL14 knockdown. (**F**) Representative western blot results of VCAM-1 and ICAM-1 in HUVECs stimulated with 10 ng/ml TNF-α for 12 h with or without METTL14 knockdown. (**G**) QRT-PCR detection of VCAM-1 and ICAM-1 in HUVECs with or without METTL14 knockdown or METTL14 overexpression. (**H**) Representative western blot results of VCAM-1 and ICAM-1 in HUVECs with or without METTL14 knockdown or METTL14 overexpression. (**I and J**) METTL14 immunostaining in atherosclerosis samples from APOE^-/-^ mice and the statistics on the proportion of METTL14-positive cells. Non AS represents the normal vascular area near the atherosclerotic plaques. AS stands for the atherosclerotic plaque area. The white and yellow arrows indicate METTL14-positive endothelial cells in the AS plaque and Non AS area, respectively. (**K**) QRT-PCR detection of METTL14 mRNA in normal vascular tissues or atherosclerotic lesions collected from APOE knockout mice. (Data are presented as the mean ± SEM. A, D, E, and G, One-way ANOVA with Bonferroni's post hoc test was applied to compare the indicated groups. J and K, Two-tailed unpaired Student's *t* test was applied to compare the two groups. *P<0.05, **P<0.01, and ***P<0.001).

At the same time, we examined whether the expression levels of m^6^A modification system-related proteins, including writers and erasers, were significantly altered after TNF-α-induced endothelial inflammation. As shown in Figure 1A, METTL14 mRNA expression was increased markedly after the stimulation of HUVECs with TNF-α for 6 and 12 h. The protein level of METTL4 was also upregulated significantly compared with the other related proteins (Figure 1B). Inflammatory stimuli increased the expression of the adhesion molecules E-selectin, VCAM-1, and ICAM-1 in endothelial cells, leading to increased adhesion of monocytes to the endothelium, which is one of the initial steps in the development of atherosclerosis. Here, we examined whether METTL14 was involved in TNF-α-induced endothelial-mononuclear adhesion. As shown in Figures 1C and 1D, METTL14 knockdown by siRNA transfection suppressed TNF-α-induced endothelial-mononuclear adhesion. Next, we detected the protein and mRNA levels of VCAM-1, ICAM-1, and E-selectin in TNF-α-induced endothelial cells with or without METTL14 knockdown. Results showed that METTL14 knockdown suppressed TNF-α-induced VCAM-1 and ICAM-1 upregulation, but had no effect on the expression of E-selectin (Figure 1E and 1F). We also examined the changes in the basal protein and mRNA levels of VCAM-1, ICAM-1, and E-selectin due to METTL14 knockdown or overexpression and found that METTL14 increased the basal expression levels of VCAM-1 and ICAM-1 (Figure 1G and 1H). To determine whether METTL14 was involved in atherosclerosis, we detected METTL14 expression in atherosclerotic samples from APOE-knockout mice. Results showed that METTL14 protein expression in atherosclerotic plaque endothelial cells was significantly higher than in non-atherosclerotic plaque endothelial cells (Figure 1I and 1J). The mRNA results were also consistent with our hypothesis (Figure 1K).

### METTL14 promotes the expression of adhesion molecules independent of m^6^A modification

**Figure 2:**
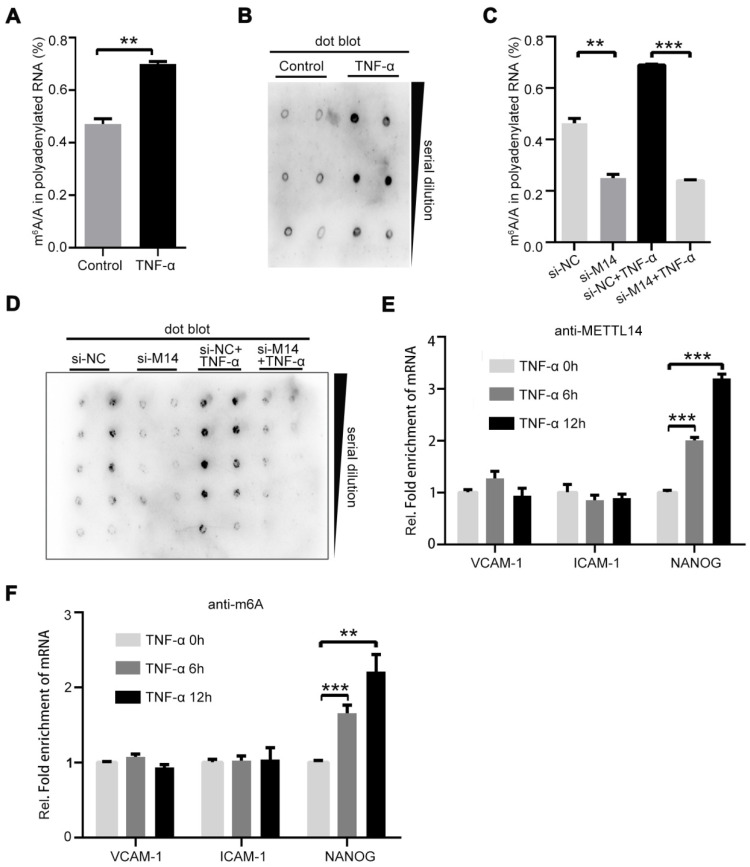
** METTL14 mediates TNF-α-induced m^6^A/A ratio upregulation in endothelial cells.** (**A**) The overall m^6^A/A ratio in polyadenylated RNA is detected using the EpiQuik^TM^ m6A RNA methylation quantification kit after HUVECs were stimulated with TNF-α (10 ng/ml, 12 h). (Data are presented as the mean ± SEM. Two-tailed unpaired Student's *t* test was applied to compare the two groups. **P<0.01, compared with the Normal tissue group). (**B**) Dot blot assay using the anti-m^6^A antibody in HUVECs stimulated with TNF-α (10 ng/ml, 12 h). (**C**) Overall m^6^A/A ratio in polyadenylated RNA is detected in HUVECs transfected with si-METTL14 after TNF-α stimulation (10 ng/ml, 12 h). (Data are presented as the mean ± SEM. One-way ANOVA with Bonferroni's post hoc test was applied to compare the indicated control groups. **P<0.01, ***P<0.001). (**D**) Dot blot assay using the anti-m^6^A antibody in HUVECs transfected with si-METTL14 after TNF-α stimulation (10 ng/ml, 12 h). (**E and F**) RIP analysis of the interaction of METTL14 or m^6^A with VCAM-1 and ICAM-1 mRNA with or without TNF-α (10 ng/ml, 6/12 h) stimulation. The enrichment of VCAM-1, ICAM-1 and NANOG mRNA with antibodies targeted against METTL14 or m^6^A was measured by qRT-PCR and normalized to the input.

It is currently believed that information on m^6^A modification is mainly “written” by the METTL3-METTL14-WTAP protein complex 25. We speculated that TNF-α treatment changes the ratio of m^6^A/A. Thus, we isolated polyadenylated RNA from endothelial cells with or without TNF-α treatment. As shown in Figure 2A, the total m^6^A/A ratio of polyadenylated RNA was increased in TNF-α-induced endothelial cells. The same results were obtained in the dot blot assay (Figure 2B). Since TNF-α induction increases METTL14 expression, we examined whether the upregulation of the total m^6^A/A ratio was mediated by METTL14 in TNF-α-induced endothelial cells. Knockdown of METTL14 suppressed the increase in total m^6^A/A ratio of polyadenylated RNA in TNF-α-induced endothelial cells (Figure 2C and 2D). Moreover, VCAM-1 and ICAM-1 mRNA levels were increased by TNF-α treatment and METTL14 overexpression; thus, we determined whether the upregulation of VCAM-1 and ICAM-1 mRNA by METTL14 overexpression was dependent on m^6^A modifications. Interestingly, TNF-α induction had no significant effect on the association between METTL14 and ICAM-1 or VCAM-1 but nevertheless promoted the binding of METTL14 to NANOG 26 (Figure 2E). RIP assay using m^6^A antibody showed similar results (Figure 2F), indicating that METTL14 promotes VCAM-1 and ICAM-1 expression independent of the m^6^A modification mechanism.

### METTL14 modifies FOXO1 mRNA to promote TNF-α-induced endothelial monocyte adhesion

**Figure 3:**
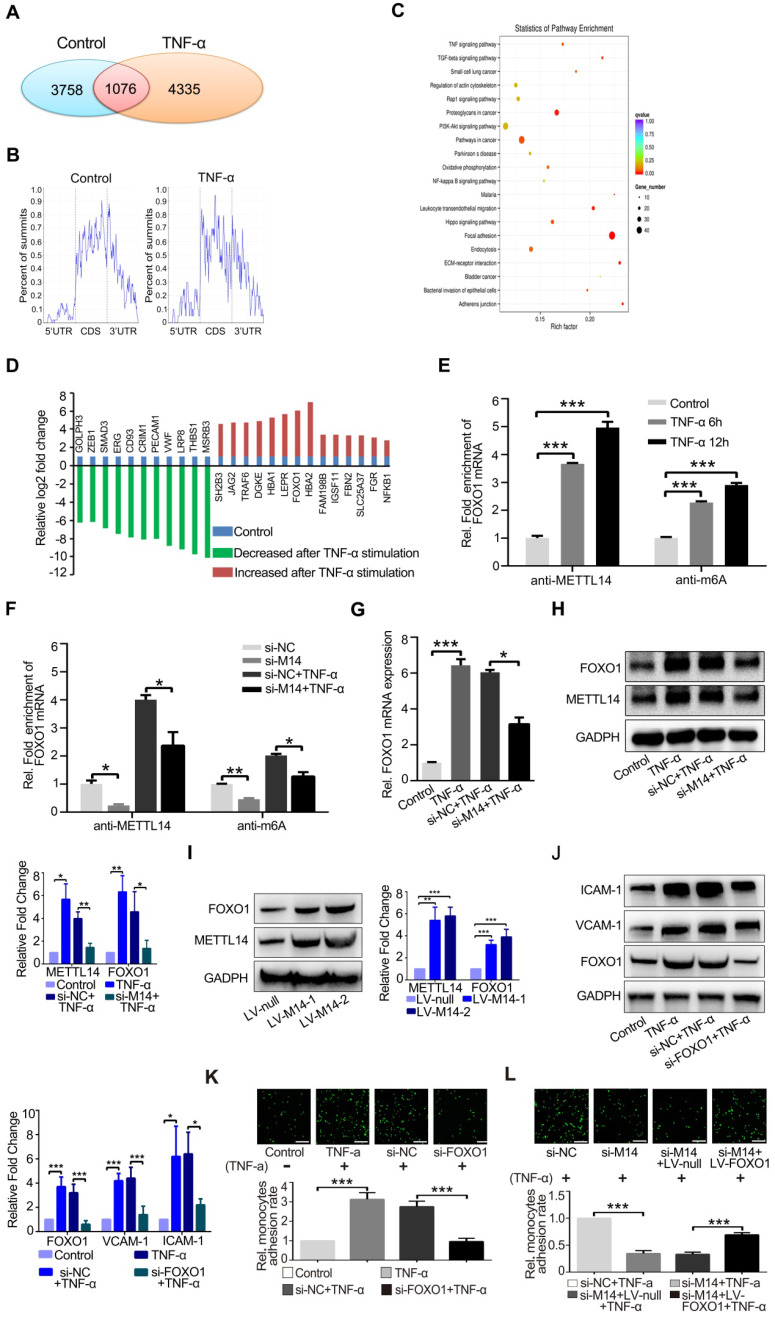
** FOXO1 mRNA is modified by METTL14 during TNF-α-induced endothelial-monocyte adhesion.** (**A**) The number of overlapping bound genes between the PBS (control) and TNF-α-stimulated groups of HUVECs. (**B**) Distribution of the m^6^A peaks across the length of the mRNAs between the control and TNF-α-stimulated groups. (**C**) GO analysis with the upregulated transcripts that were covered by a unique peak. The cutoff parameters for enrichment analysis using Cytoscape software are as follows: *p* < 0.005, FDR: *q* < 0.1, and overlap cutoff: > 0.5. (**D**) Potential mRNAs that were significantly up- or down-regulated with m^6^A modification after TNF-α stimulation. (**E**) RIP analysis of the interaction of METTL14 or m^6^A with FOXO1 mRNA with or without TNF-α stimulation (10 ng/ml, 6/12 h). The enrichment of FOXO1 mRNA was measured by qRT-PCR with antibodies targeted against METTL14 or m^6^A and normalized to the input. (**F**) RIP analysis of the interaction of METTL14 or m^6^A with FOXO1 mRNA with or without si-METTL14 transfection and TNF-α stimulation (10 ng/ml, 6/12 h). The enrichment of FOXO1 mRNA m^6^A was measured by qRT-PCR with antibodies against METTL14 or and normalized to the input. (**G**) RT-PCR detection of FOXO1 mRNA expression with or without si-METTL14 transfection and TNF-α stimulation (10 ng/ml, 12 h). (**H**) Representative western blot results and its relative expression fold change of FOXO1 in HUVECs stimulated with 10 ng/ml TNF-α for 12 h with or without METTL14 knockdown. (**I**) Representative western blot results showing the relative fold change of FOXO1 expression in HUVECs transfected with METTL14 overexpression lentivirus (LV-M14-1, MOI=10 and LV-M14-2, MOI=20). (**J**) Representative western blot results showing the relative fold change of VCAM-1 and ICAM-1 expression in HUVECs stimulated with 10 ng/ml TNF-α for 12 h with or without FOXO1 knockdown. (**K**) Endothelial-monocyte adhesion assay in HUVECs after knockdown of FOXO1 (with or without TNF-α stimulation) and the relative monocyte adhesion rate. (**L**) Endothelial-monocyte adhesion assay in HUVECs after METTL14 knockdown or FOXO1 overexpression (with or without TNF-α stimulation) and the relative monocyte adhesion rate. (E-J) Data are presented as the mean ± SEM. One-way ANOVA with Bonferroni's post hoc test was applied to compare the indicated groups. (K and L) Two-tailed unpaired Student's *t*-test was applied to compare the indicated two groups. *P<0.05, **P<0.01, and ***P<0.001). (K and L) Three independent experiments were performed; magnification, ×100.

To determine the mechanism by which METTL14 regulates VCAM-1 and ICAM-1-mediated endothelial monocyte adhesion, we used an antibody-based method that recognizes m^6^A. Immunoprecipitation (IP) of m^6^A-modified RNA collected from control and TNF-α-stimulated endothelial cell samples, followed by high-throughput RNA sequencing (MeRIP-seq) identified 3,758 methylated (m^6^A modification) fragments in the control group and 4,335 in the TNF-α group, with 1,076 overlapping sites (Figure 3A). m^6^A RNA predominantly occurred in the protein-coding RNAs found in both the control and TNF-α groups (Figure 3B), thus supporting previous observations that m^6^A methylation occurs primarily in protein-coding transcripts 27, 28. Gene ontology (GO) analysis of these upregulated genes showed that the transcripts were mainly involved in specific focal adhesion, adhesion junctions, as well as the TNF and PI3K-Akt signaling pathways, which play important roles in endothelial inflammation (Figure 3C). Finally, we screened the mRNAs that were significantly up- or down-regulated due to m^6^A modification after TNF-α stimulation (Figure 3D). For further analysis of the MeRIP-seq results, we initially focused on the transcription factor FOXO1, as previous studies have shown that FOXO1 can promote transcription by directly binding to the promoter regions of VCAM-1 and ICAM-1^29^-^31^. As shown in Figure 3E, TNF-α stimulation increased the association between METTL14 and FOXO1 mRNA, as well as promoted FOXO1 mRNA m^6^A modification. In addition, knockdown of METTL14 by siRNA transfection decreased the m^6^A modification on FOXO1 mRNA and the binding of METTL14 to FOXO1 mRNA (Figure 3F).

Next, we detected the protein levels of FOXO1 after TNF-α stimulation and METTL14 knockdown. Results showed that TNF-α promoted the upregulation of FOXO1 total protein and nuclear distribution (Figure S2A), whereas METTL14 knockdown partly attenuated this upregulation at both mRNA and protein levels (Figure 3G and 3H). In addition, our results showed that the overexpression of METTL14 significantly increased the expression of FOXO1 (Figure 3I). The above results imply that METTL14-mediated FOXO1 mRNA m^6^A modification promotes FOXO1 upregulation in TNF-α-induced endothelial cells.

To verify the role of FOXO1 in TNF-α-induced VCAM-1 and ICAM-1 expression, we performed FOXO1 knockdown by siRNA transfection (Figure S2B). Results showed that the protein levels of VCAM-1 and ICAM-1 were decreased (Figure 3J) and TNF-α-induced increase in endothelial-monocyte adhesion was significantly reduced (Figure 3K). Moreover, METTL14 ectopic overexpression increased endothelial-monocyte adhesion (Figure S2C) and FOXO1 overexpression rescued the METTL14 knockdown-induced decrease in endothelial-monocyte adhesion (Figure 3L). Therefore, we believe that TNF-α-induced increase in VCAM-1 and ICAM-1 expression levels was mediated by METTL14 and that endothelial-monocyte adhesion may be achieved by modulating FOXO1 expression. Since FOXO1-Sirt1 interaction is well-recognized, we detected the expression of Sirt1 and found that TNF-α stimulation upregulated Sirt1 protein expression (Figure S2D).

### YTHDF1 promotes FOXO1 mRNA translation by recognizing the m^6^A-modified site

**Figure 4:**
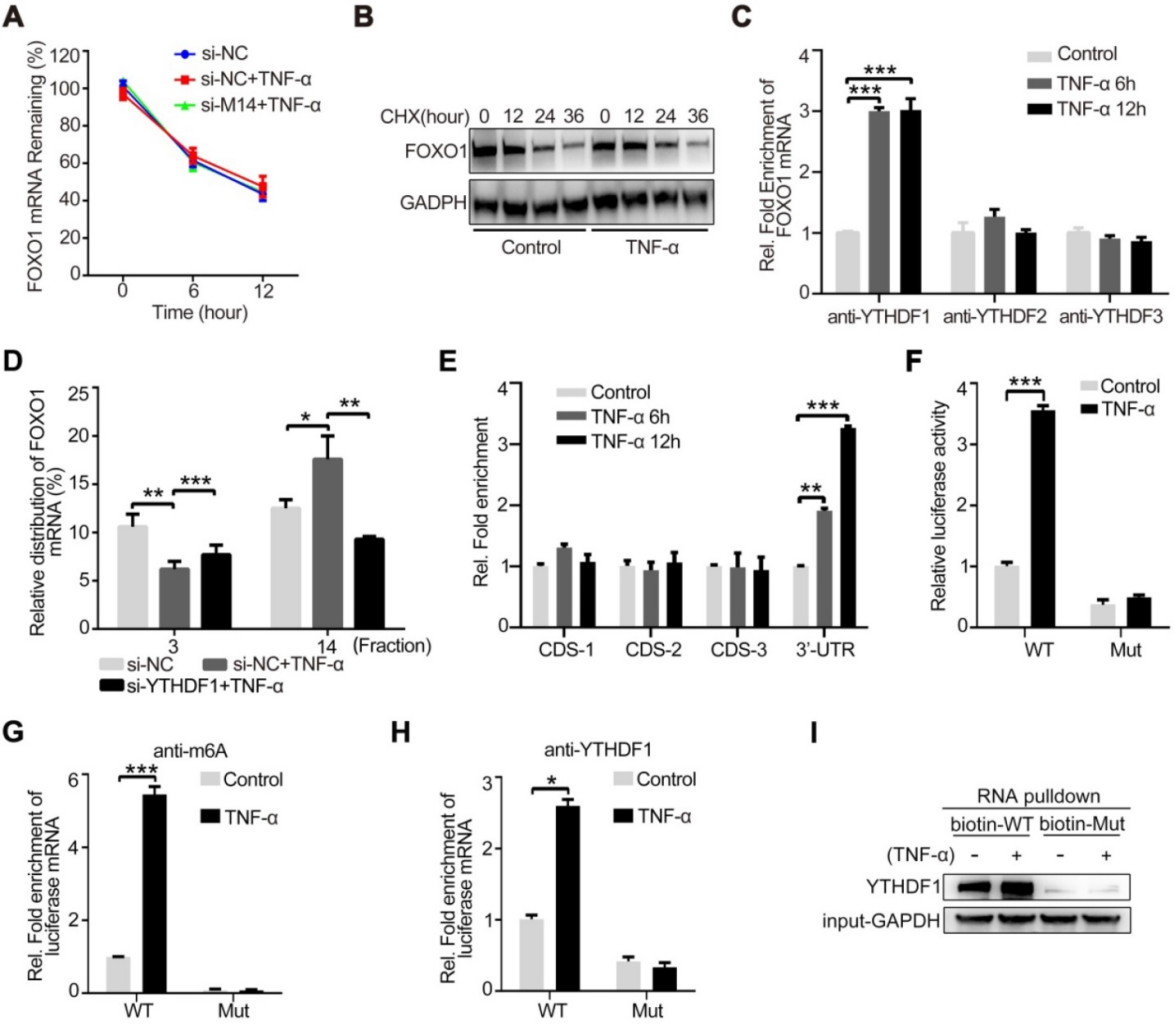
** YTHDF1 enhances FOXO1 translation by recognizing the m^6^A modification site on the 3ʹ-UTR of FOXO1 mRNA.** (**A**) The expression of FOXO1 mRNA was measured using qRT-PCR after si-METTL14 transfection of HUVECs with or without TNF-α (10 ng/ml, 12 h) stimulation and later treated with ActD (4 µM). (**B**) The stability of the FOXO1 protein was measured by western blotting after HUVECs were treated with or without TNF-α (10 ng/ml, 12 h). Cells were then treated with CHX (10 µM). (**C**) RIP analysis of the interaction of YTHDF1/2/3 with FOXO1 mRNA with or without TNF-α stimulation (10 ng/ml, 6/12 h). The enrichment of FOXO1 mRNA was measured by qRT-PCR with antibodies against YTHDF1, YTHDF2, or YTHDF3 and normalized to the input. (**D**) qRT-PCR analysis of FOXO1 mRNA in HUVECs after TNF-α stimulation (10 ng/ml, 12 h) with or without si-YTHDF1 transfection and fractioned into polysomes (fraction 3 and 14 were selected). (**E**) RIP analysis of the interaction of m^6^A with different suspected m^6^A regions of FOXO1 mRNA (CDS-1, CDS-2, CDS-3, and 3ʹ-UTR) with or without TNF-α stimulation (10 ng/ml, 6/12 h). The enrichment of the suspected m^6^A regions was measured by qRT-PCR with an antibody against m^6^a and normalized to the input. (**F**) Luciferase activity of HUVECs transfected with WT or mutant plasmids with or without TNF-α stimulation (10 ng/ml, 12 h) was measured using the dual luciferase reports system. (**G**) RIP analysis of the interaction of m^6^A with WT or mutant plasmids in HUVECs with or without TNF-α stimulation (10 ng/ml, 12 h). The enrichment of the suspected m^6^A regions was measured by qRT-PCR with an antibody against m^6^A and normalized to the input. (**H**) RIP analysis of the interaction of YTHDF1 with WT or mutant plasmids in HUVECs with or without TNF-α stimulation (10 ng/ml, 12 h). The enrichment of the suspected m^6^A regions was measured by qRT-PCR with an antibody against m^6^A and normalized to the input. (**I**) RNA pulldown assay of biotin-tagged WT or mutant plasmids cultured with HUVEC lysates (with or without TNF-α stimulation). Data are presented as the mean ± SEM. (A, C, D, and E) One-way ANOVA with Bonferroni's post hoc test was applied to compare the indicated groups. (F, G, and H) Two-tailed unpaired Student's *t*-test was applied to compare the indicated two groups. *P<0.05, **P<0.01, and ***P<0.001).

The main mechanism of action by which m^6^A modification regulates protein expression is via the regulation of mRNA stability, shearing, nuclear transport, and translation 8. Among these, m^6^A modification most commonly regulates the stability of mRNA or promotes its translation. Therefore, we first examined the mRNA stability of FOXO1. As shown in Figure 4A, TNF-α stimulation and METTL14 knockdown had no significant effect on the stability of FOXO1 mRNA. However, after treatment with CHX to stop translation, TNF-α-induced FOXO1 upregulation was attenuated, indicating that this effect of TNF-α is dependent on translation (Figure 4B). Since m^6^A information is 'written', further recognition of the 'reader' proteins, including YTHDF1, YTHDF2, and YTHDF3, is required to determine the final regulatory effect. To detect the binding of FOXO1 mRNA to the m^6^A 'reader' proteins, we performed RIP assay in TNF-α-stimulated endothelial cells and found that YTHDF1 can directly bind to the FOXO1 mRNA (Figure 4C). This suggests that METTL14 may enhance FOXO1 mRNA translation through YTHDF1. Through the polysome fractionation assay (selected fraction 3 and 14 for analysis) we confirmed that TNF-α stimulation significantly enhanced the translation ability of FOXO1 mRNA, and this effect was significantly inhibited after YTHDF1 knockdown (Figure 4D). Next, we searched the concrete m^6^A sites and identified four suspected sites (Table S1). RIP assay results showed that the 3ʹ-UTR site is the real m^6^A modification site (Figure 4E). To confirm this results, we constructed pMIR-REPORT plasmids containing the wild-type (GGACU) or mutant (GGCCU) sites. By detecting luciferase fluorescence, we found that TNF-α stimulation increased the luciferase activity of wild type (WT) plasmids but had no effect on the mutant plasmids (Figure 4F). Me-RIP assay also showed that TNF-α stimulation promoted m6A modification of WT plasmids but had no effect on the mutant plasmids (Figure 4G). Furthermore, we also detected the association between YTHDF1 and the related region. Results showed binding of YTHDF1 to the WT region, which was strengthened by TNF-α stimulation; however, this effect was not observed in the mutated region (Figure 4H). Further, RNA pull-down assay using biotin-tagged WT or mutant regions confirmed the binding between YTHDF1 and the WT region (Figure 4I). Hence, our results suggest that TNF-α-induced endothelial cell inflammation enhances m^6^A modification on FOXO1 mRNA and its translation through YTHDF1 recognition.

### METTL14 cooperates with FOXO1 to promote VCAM-1 and ICAM-1 transcription

**Figure 5:**
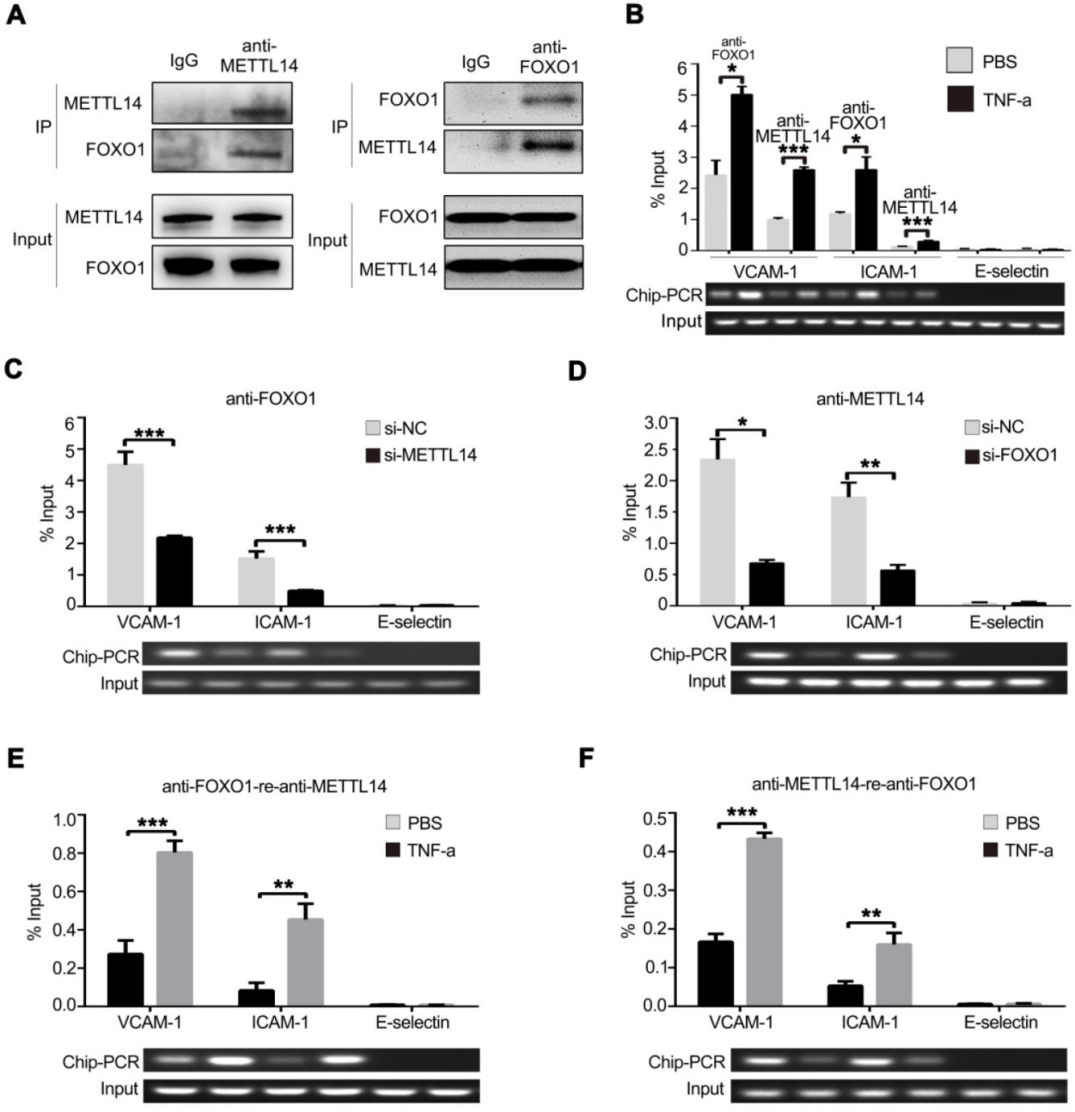
** METTL14 cooperates with FOXO1 to promote VCAM-1 and ICAM-1 transcription.** (**A**) HUVECs were lysed and precipitated using anti-METTL14 or anti-FOXO1 antibodies, followed by immunoblot analyses with indicated antibodies. (**B**) ChIP assay showed that the ability of METTL14 and FOXO1 proteins to bind the VCAM-1 and ICAM-1 promoters was significantly increased in TNF-α-stimulated HUVECs, while there was no change in their ability to bind the E-selectin promoter. (**C and D**) ChIP assay demonstrated the effect of METTL14 knockdown on the binding of FOXO1 to VCAM-1, ICAM-1, or E-selectin promoter regions, as well as the effect of FOXO1 knockdown on the binding of METTL14 to VCAM-1 and ICAM-1 promoter regions. (**E and F**) CHIP-re-CHIP results showed that the binding of the METTL14-FOXO1 complex to the promoter regions of VCAM-1 and ICAM-1 was significantly increased after TNF-α stimulation of HUVECs. (B-F) Data are presented as mean ± SEM. One-way ANOVA with Bonferroni's post-hoc test was applied to compare the indicated two groups. *P<0.05 and **P<0.01.

To investigate whether METTL14 promotes VCAM-1 and ICAM-1 transcription partly independent of its enzymatic activity, the endogenous METTL14 protein complex was precipitated from HUVECs and subjected to mass spectrometry analysis. Results showed that FOXO1 also binds METTL14 in HUVECs (Figure S3A). Next, we ectopically expressed flag-tagged METTL14 and myc-tagged FOXO1 in 293T cells and performed co-IP using anti-flag or anti-myc antibodies. As shown in Figure S3B, flag-tagged METTL14 interacted with myc-tagged FOXO1 in 293T cells. Furthermore, we performed endogenous co-IP in HUVECs using anti-METTL14 or anti-FOXO1 antibodies and obtained the same results (Figure 5A). Because METTL14 interacts with FOXO1, we wanted to determine whether METTL14 participates in FOXO1-mediated VCAM-1 and ICAM-1 transcription. ChIP analysis showed that TNF-α stimulation promoted METTL14 or FOXO1 binding to the VCAM-1 and ICAM-1 promoters but not the E-selectin promoter (Figure 5B). We then evaluated the significance of METTL14 and FOXO1 interaction in TNF-α-induced VCAM-1 and ICAM-1 transcription. As shown in Figure 5C and 5D, METTL14 knockdown suppressed the binding of FOXO1 to the VCAM-1 and ICAM-1 promoters, and FOXO1 knockdown inhibited the binding of METTL14 to the VCAM-1 and ICAM-1 promoters. To detect the direct interaction of FOXO1 and METTL14 with the VCAM-1 and ICAM-1 promoters, we further performed ChIP-re-ChIP analysis. Results showed that TNF-α treatment induced the binding of the FOXO1-METTL14 complex to the VCAM-1 and ICAM-1 promoters (Figure 5E and 5F). These results indicate that METTL14 mediates the binding of FOXO1 to the VCAM-1 and ICAM-1 promoters to activate transcription.

### Decreased METTL14 expression significantly inhibits atherosclerosis *in vivo*

**Figure 6:**
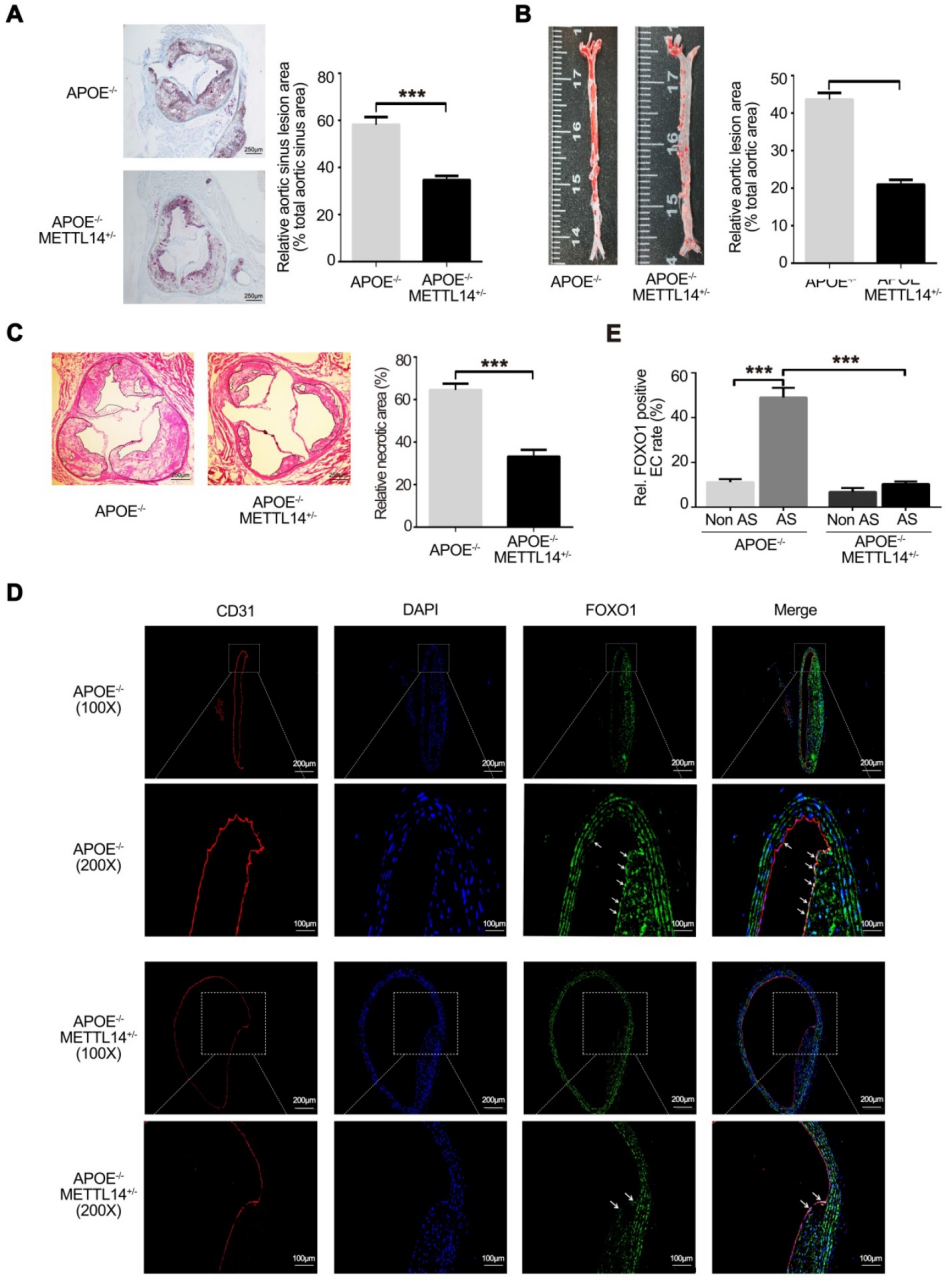
** METTL14 knockout can significantly inhibit atherosclerosis development.** (**A**) METTL14^+/-^/APOE^-/-^ and control APOE^-/-^ mice were fed WD for 12 weeks. Representative images and quantification of the aortic root lesion area stained with oil red O are shown. (n=10-12 for each group). (**B**) Representative images and quantification of the aorta en face lesion stained with oil red O (n=10-12 for each group). (**C**) Necrotic core area (H&E) in the aortic root (n=10 per group). (**D and E**) Immunofluorescence staining showing the expression of FOXO1-positive cells in the atherosclerotic plaque and non-plaque regions of METTL14^+/-^/APOE^-/-^ and APOE^-/-^ mice (n=10 per group). All representative images are from mice fed WD. (A, B, C, and E) Data are presented as mean ± SEM. (E) Two-tailed unpaired Student's *t*-test was applied to compare the indicated two groups. (A, B, and C) One-way ANOVA with Bonferroni's post-hoc test was applied to compare the indicated groups. (***P<0.001).

To further investigate the effects of METTL14 on atherosclerosis *in vivo*, METTL14 knockout mice were used. Previous studies have shown that METTL14 gene knockout causes death in mice during the embryonic period 32, 33. Therefore, we used METTL14 heterozygous gene knockout mice (METTL14^+/-^) for further research. The results of genotyping are shown in Supplementary Figure 4A and 4B. Simultaneously, we assessed the expression of METTL14 and other related molecules in METTL14^+/-^ and WT mice. Results showed that METTL14, ICAM-1, and VCAM-1 expression was significantly lower in the vascular tissues of METTL14^+/-^/APOE^-/-^ mice than in the tissues of WT mice, while YTHDF1 expression showed no difference among both groups (Figure S4C and S4D). Next, METTL14^+/-^/APOE^-/-^ mice and APOE^-/-^ mice (Control group) were fed a western diet (WD) for 12 weeks. No differences in body weight and plasma lipid profiles were observed between the two groups, indicating that METTL14 deficiency does not have a major impact on weight gain or lipid metabolism (Table S2). After 12 weeks of WD, quantification of the lesion areas in the aortic roots revealed significantly smaller lesions in METTL14^+/-^/APOE^-/-^ mice than in APOE^-/-^ mice (Figure 6A). En face aorta analysis showed that the lesion area was significantly smaller in METTL14^+/-^/APOE^-/-^ mice than in APOE^-/-^ mice (Figure 6B). Furthermore, the necrotic core area was also markedly reduced in METTL14^+/-^/APOE^-/-^ mice compared with that in APOE^-/-^ mice (Figure 6C). These results suggest that METTL14 gene knockout can significantly reduce the development of atherosclerosis. Additionally, we collected vessel samples from METTL14^+/-^/APOE^-/-^ mice and APOE^-/-^ mice for immunofluorescence staining. Results showed that FOXO1 expression in the atherosclerotic plaque area of APOE^-/-^ mice was significantly higher than that in the non-plaque area. Furthermore, FOXO1, ICAM-1, and VCAM-1 expression in endothelial cells was significantly lower in the atherosclerotic plaque area of METTL14^+/-^/APOE^-/-^ mice than in the plaque area of APOE^-/-^ mice (Figure 6D, 6E, and Figure S5A-G). Hence, our results confirmed that METTL14 may inhibit atherosclerosis development by regulating FOXO1 expression in mice. These findings are consistent with our cytological results, which showed that METTL14 mediates TNF-α-induced inflammatory adhesion molecule expression by regulating FOXO1.

## Discussion

RNA modifications occur not only in noncoding RNAs, such as tRNAs, lncRNAs, and rRNAs, but also in mRNAs. Among more than 150 modified RNA nucleotide variants identified, the m^6^A modification is the most prevalent chemical mark in eukaryotic mRNAs; it is reversible and affects more than 7,000 mRNAs in the individual transcriptomes of mammalian cells 27. With the availability of m^6^A-specific antibodies and development of deep sequencing technology, m^6^A modification has been widely studied 15, 34. The m^6^A modification is achieved by on the action of a large protein enzyme complex. Biochemical and structural researches suggest that this protein complex mainly includes METTL3/METTLl4, in addition to certain auxiliary proteins, such as WTAP and KIAA1429, which are required for nuclear localization 35. METTL3 and METTL14 belong to a large and conserved family of methyltransferases and contain an MT-A70 domain (also known as methyltransferase domain, MTD) that catalyzes the transfer of methyl groups to adenosine bases. The two MTDs, METTL3 and METTL14, bind tightly to each other and form a catalytic center for adenine methylation. Previous studies have shown that METTL3 is the main enzyme that catalyzes the m^6^A modification, while the catalytic center of METTL14 is degenerated and does not participate in chemical reactions. Meanwhile, METTL14 is essential for METTL3/14 complex activity because it has degenerate active sites and plays a crucial structural role in maintaining complex integrity and RNA substrate binding activity 35-37. This suggests that METTL14 has an important regulatory role in m^6^A modification. Moreover, recent studies have shown that METTL14 plays vital roles in the regulation of m^6^A modification in target mRNAs in various biological processes, such as microRNA processing, viral-associated tumorigenesis, stem cell differentiation, and renal ischemic reperfusion 38-41. When the m^6^A information of mRNA is 'written', further recognition requires the 'reader' proteins. Among the m^6^A 'readers', the cytoplasmic proteins YTHDF1, YTHDF2, and YTHDF3 have been shown to directly bind and recognize m^6^A through their carboxyl-terminal YTH domain. YTHDF2-mediated degradation regulates the lifetime of target transcripts, whereas promotion of YTHDF1-mediated translation enhances translation efficiency, thus facilitating effective protein production from dynamic transcripts marked with m^6^A modification. YTHDF3 promotes protein synthesis in synergy with YTHDF1 and affects methylated mRNA decay mediated through YTHDF2.

In our study, we found that the METTL14 expression rather than METTL3 expression was significantly upregulated in TNF-α-induced endothelial cell inflammation, suggesting that although METTL3 and METTL14 have synergistic effects, they play different regulatory roles in m^6^A modification. Furthermore, m6A modifications on FOXO1 mRNA by METTL14 were recognized by YTHDF1, and FOXO1 mRNA translation was promoted. More importantly, we found that METTL14 knockout significantly inhibited atherosclerosis development, demonstrating the potential of METTL14 in the treatment of atherosclerosis and related disorders.

As an important transcription factor, the FOXO1 protein is a key regulator of cell metabolism. FOXO1 gene knockout has been shown to cause serious abnormalities in the vascular network structure, leading to the death of animal embryos, thus demonstrating the importance of FOXO1 in the process of angiogenesis 42. The role of FOXO1 protein in the maintenance of vascular homeostasis after birth has also been confirmed. Moreover, specific knockdown of FOXO1 in adult mouse vascular endothelial cells can lead to excessive proliferation and a marked decrease in apoptosis 43.

Meanwhile, endothelial cell-specific FOXO1 gene knockout mice were shown to display significantly lesser atherosclerotic plaques than wild-type mice 44. This is consistent with our findings, which demonstrated that the expression of FOXO1 in METTL14 knockdown mice was reduced and the development of atherosclerosis was significantly inhibited. Previous research also indicates that FOXO1 can inhibit the synthesis of endothelial nitric oxide synthase and promote the production of inducible NOS, leading to peroxynitrite deposition and endothelial dysfunction 45, 46. Additionally, FOXO1 expression in the endothelial cells of unstable plaques was shown to be significantly increased, promoting the dysfunction of vascular endothelial cells in the plaques and reducing the stability of atherosclerosis plaques, which caused plaque rupture, leading to acute thrombotic events, including acute coronary syndrome 47. These data indicate that FOXO1 plays an important role in the occurrence, development, and plaque instability of atherosclerosis.

VCAM-1, ICAM-1, and E-selectin are representative endothelial inflammatory adhesion molecules that are present in low levels under normal physiological conditions. When endothelial cells are exposed to various stimuli, especially inflammatory factors, their expression is significantly upregulated. The adhesion molecules play an important role in endothelial-monocyte adhesion and thus, promote the development of atherosclerosis 48, 49. Previous studies have shown that FOXO1 regulates the expression of VCAM-1, ICAM-1, and E-selectin. Among them, FOXO1 can directly bind to the promoter regions of VCAM-1 and ICAM-1 to promote transcription 29, 31, 50. The detailed mechanism underlying the upregulation of E-selectin by FOXO1 has not yet been clarified. In our study, we found that METTL14 enhances FOXO1 expression by increasing the translational capacity of FOXO1 mRNA through the m^6^A modification, and then upregulating VCAM-1 and ICAM-1 expression by direct binding to their promoter regions. These results are consistent with those of previous studies. Additionally, our results showed that METTL14 interacts with FOXO1, and this interaction promotes the transcriptional regulation of FOXO1 on VCAM-1 and ICAM-1. This finding highlights the role of METTL14 independent of the m^6^A modification.

However, this study has certain limitations. The number of human atherosclerotic samples collected were not sufficient, and further validation of the results of cytology and animal experiments in humans is necessary. Our biological sample bank collected atherosclerotic plaques from patients with carotid artery exfoliation. Follow-up studies are required to further examine the regulatory effect of METTL14 on FOXO1 in human samples. Moreover, since METTL14^+/-^ mice showed expression of METTL14 in endothelial cells, the role of METTL14 in atherosclerosis development needs further clarification. Hence, an endothelial-specific METTL14 knockout mouse model should be established in the future.

In summary, our study explores the regulatory mechanisms of endothelial cell inflammation from an epigenetic perspective. Our findings confirmed that METTL14 can enhance its translation by increasing m^6^A modification on FOXO1, thereby increasing adhesion molecule expression, mediating endothelial-monocyte adhesion, and participating in atherosclerosis development. Furthermore, we also demonstrated that METTL14 acts as a protein independent of m^6^A modification by interacting with FOXO1 and enhancing the FOXO1-mediated transcription of adhesion molecules. To the best of our knowledge, this is the first study on m^6^A modifications in atherosclerosis, which demonstrated the effects of METTL14 on endothelial inflammation and atherosclerosis both *in vivo* and *in vitro*. Hence, our findings may provide new ideas for the prevention and treatment of atherosclerosis.

## Supplementary Material

Supplementary figures and tables.

## Figures and Tables

**Figure 7:**
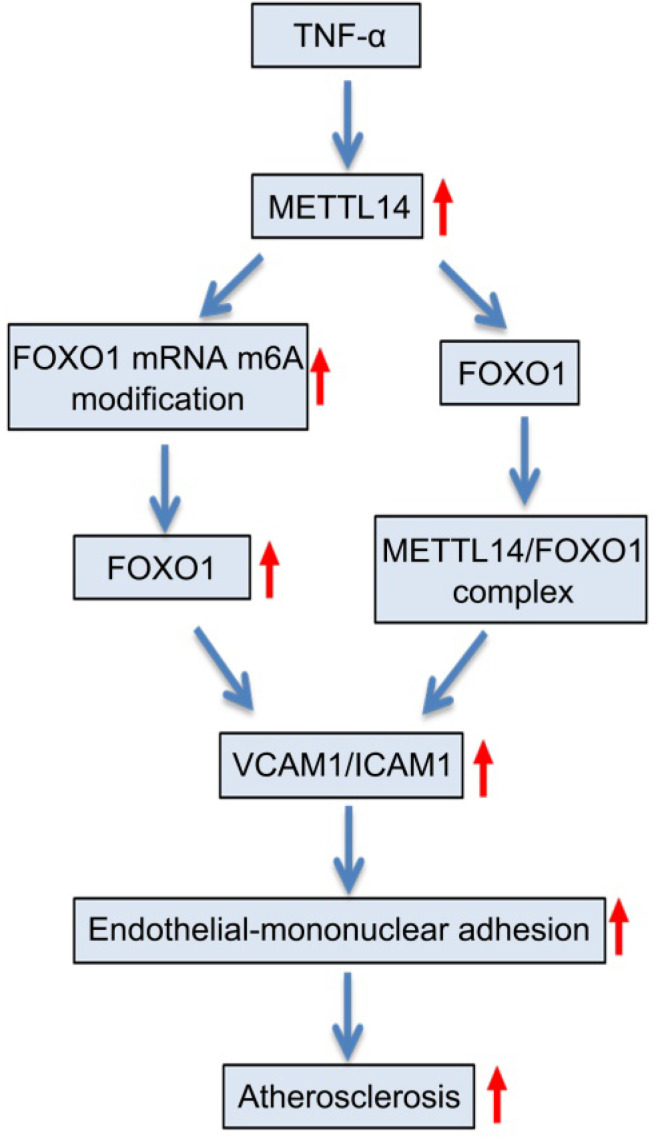
** Proposed scheme of the mechanism of METTL14-mediated m^6^A modification affects endothelial inflammation and atherosclerosis by regulating FOXO1.** The expression of METTL14 was increased in endothelial cells stimulated by TNF-α, which enhanced the m^6^A modification of FOXO1 mRNA and promoted the expression of FOXO1, and thus promoting the expression of adhesion molecules. At the same time, METTL14 can also interact with FOXO1 to promote the expression of adhesion molecules, which in turn mediates TNF-α-induced endothelial inflammation and atherosclerosis.
