# Supplementary material for: METTL14 aggravates endothelial inflammation and atherosclerosis by increasing FOXO1 N6-methyladeosine modifications
Source: Theranostics. 2020 Jul 11;10(20):8939–56. doi: 10.7150/thno.45178 (PMC7415798; doi:10.7150/thno.45178)
Supplement: Supplementary file 1 — Supplementary figures and tables. [file thnov10p8939s1.pdf]

Supplement Figure 1: TNF-α-induced adhesion molecule expression in endothelial cells.

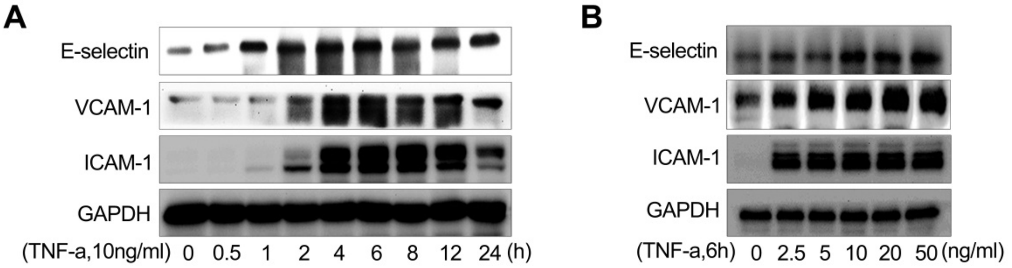

(A) Representative images and quantification of expression of endothelial adhesion molecules (VCAM-1, ICAM-1, and E-selectin) after treatment of HUVECs with 10 ng/ml of TNF-α at different time points.

(B) Representative images and quantification of expression of endothelial adhesion molecules (VCAM-1, ICAM-1, and E-selectin) in HUVECs treated with different concentrations of TNF-α for 6 h.

Supplement Figure 2: TNF-α-induced FOXO1 expression in endothelial cells.

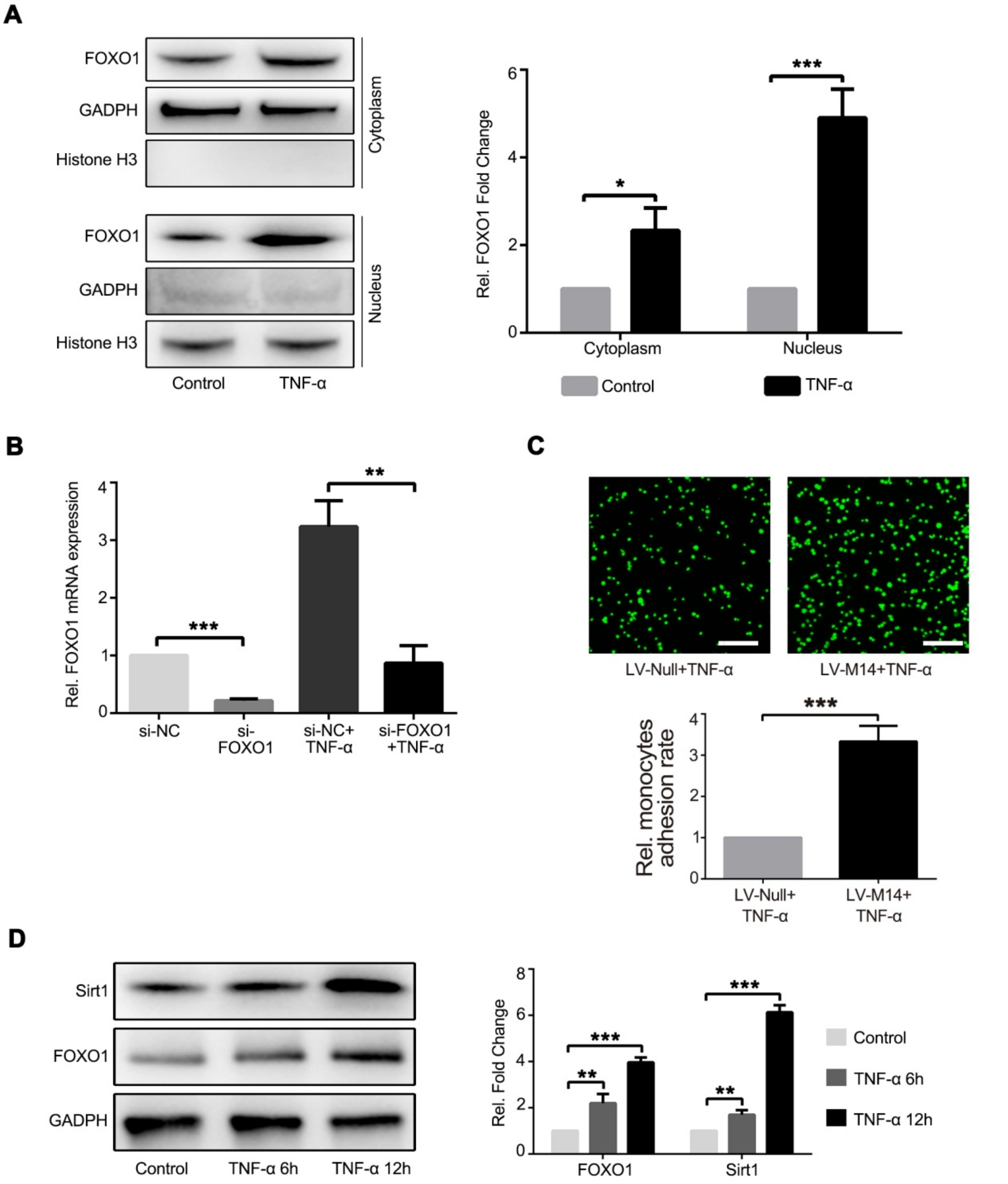

(A) Representative images and quantification of FOXO1 expression in the cytoplasm and nucleus after treatment of HUVECs with TNF-α (10 ng/ml, 12 h).

(B) The effect of FOXO1 knockdown on FOXO1 mRNA expression with or without TNF-α stimulation (10 ng/ml, 12 h).

(C) The effect of overexpression of METTL14 on TNF-α-induced monocyte-endothelial cell adhesion.

(D) Representative images and quantification of FOXO1 and Sirt1 expression after

treatment of HUVECs with TNF-α (10 ng/ml, 6/12 h).

(A, B, and C) Data are presented as mean ± SEM. Two-tailed unpaired Student's *t*-test was applied to compare the indicated two groups. (D) One-way ANOVA with Bonferroni's post-hoc test was applied to compare the indicated groups. *$P<0.05$, **$P<0.01$, and ***$P<0.001$.

Supplement Figure 3: METTL14 cooperates with FOXO1 to promote VCAM-1 and ICAM-1 transcription.

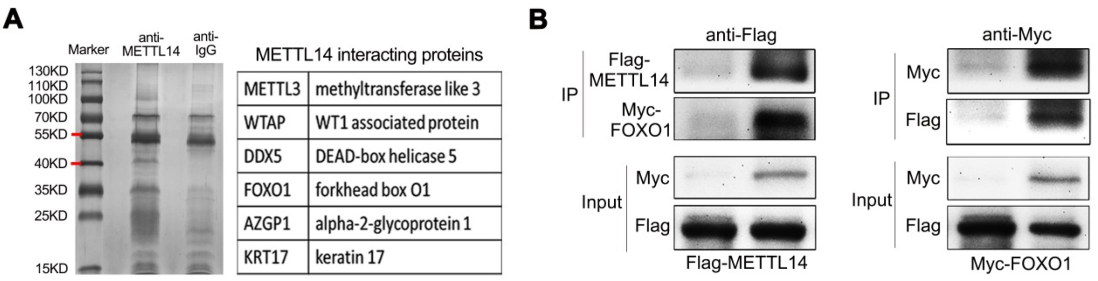

(A) The protein complex was precipitated with anti-METTL14 antibody using IgG as an endogenous control. The METTL14-interacting proteins are listed in the Table.

(B) 293T cells were transfected with flag-METTL14 and myc-FOXO1 and co-IP was performed using anti-flag or anti-myc antibody, followed by immunoblot analysis with indicated antibodies.

Supplement Figure 4: Genotype and knockdown verification of METTL14 knockout mice and related gene expression profile.

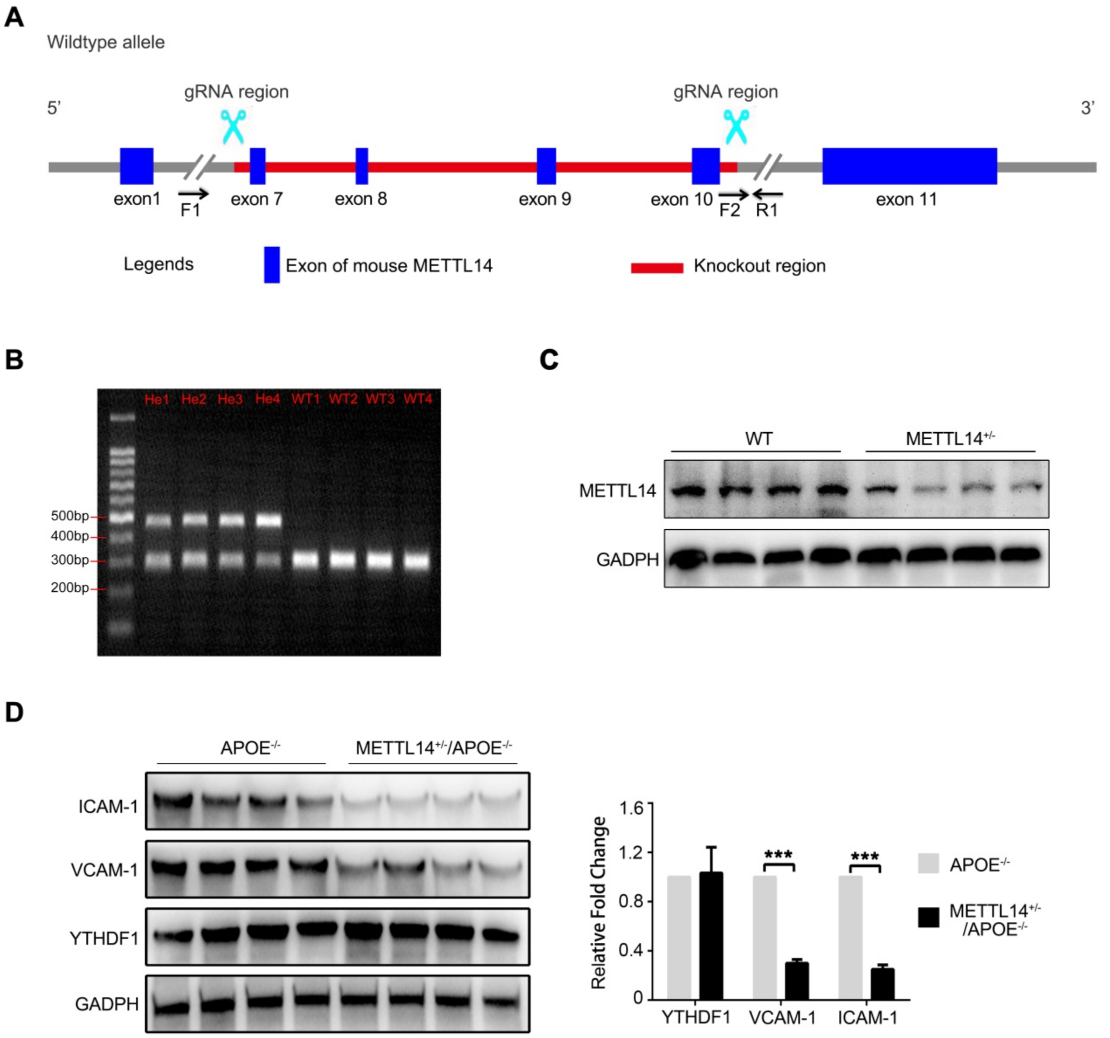

(A) An overview of the targeting strategy used to construct the METTL14 knockout mouse model. METTL14 knockout was performed using CRISPR/Cas9-based targeting strategy. Two gRNAs were designed between the 7th and 10th exon, and the sequence was deleted. Two pairs of PCR primers were used to identify whether the mice were heterozygous: the F1/R1 primer can amplify a 474 bp sequence and the F2/R1 primer can amplify a 247 bp sequence, thus confirming heterozygous status.

(B) Genotype verification of METTL14 knockout mice. Two bands of 437 and 274 bp appeared in METTL14 knockout heterozygous mice (He 1-4), while only one band of 274 bp was seen in METTL14 wild-type mice (WT 1-4).

(C) METTL14 expression in the vascular tissues of METTL14$^{+/-}$ and C57BL/6 mice.

(D) Representative images and quantification of ICAM-1, VCAM-1, and YTHDF1 expression in the vascular tissues of $APOE^{-/-}$ and $METTL14^{+/-}/APOE^{-/-}$ mice. Data are presented as mean ± SEM. Two-tailed unpaired Student's *t*-test was applied to compare the indicated two groups. ***$P<0.001$.

Supplement Figure 5: Immunofluorescence staining of METTL14, VCAM-1 and ICAM-1 positive cells in the atherosclerotic plaque regions of METTL14$^{+/-}$/APOE$^{-/-}$ and APOE$^{-/-}$ mice.

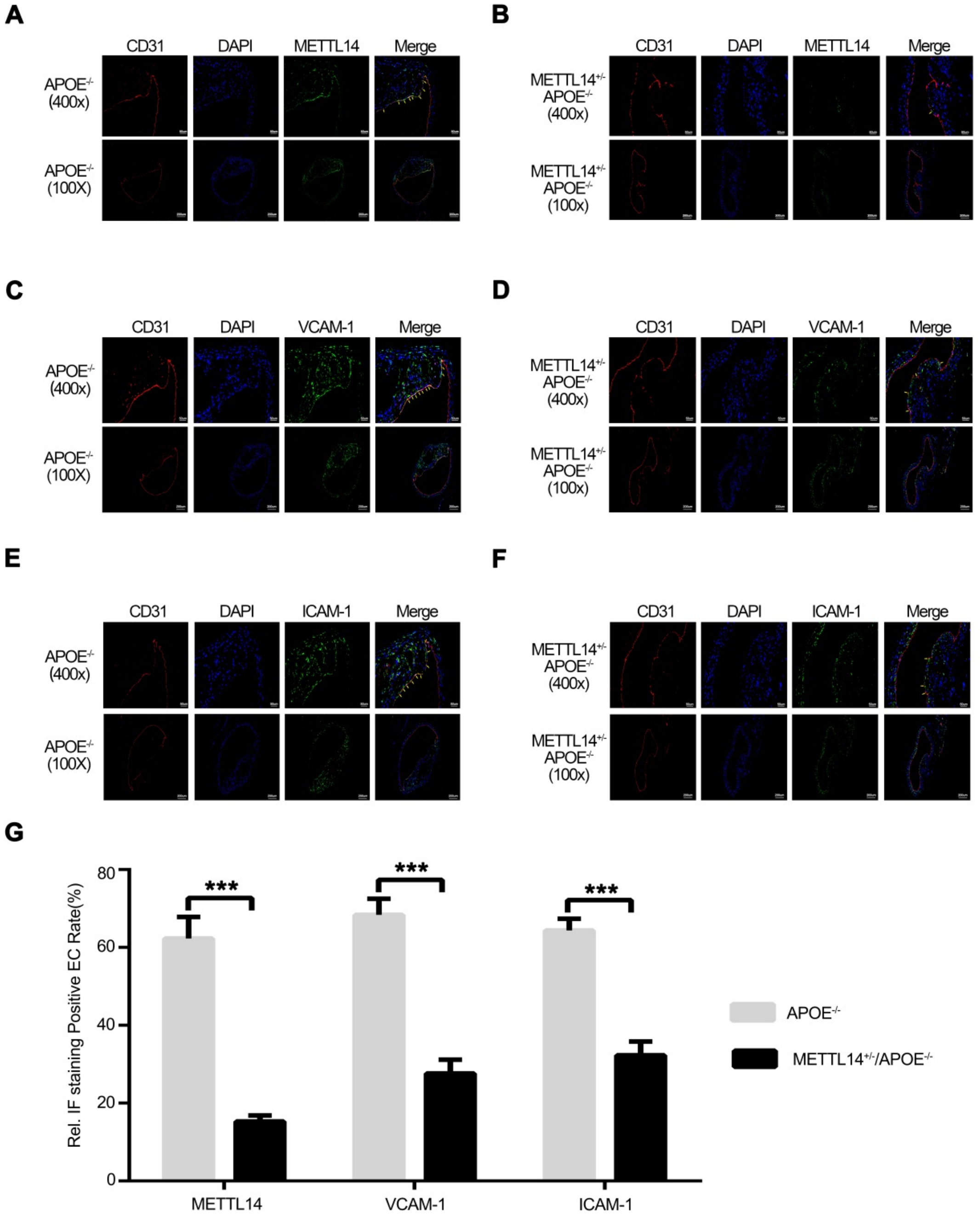

(A to G) Immunofluorescence staining showing the expression of METTL14-, VCAM-1-, and ICAM-1-positive cells in the atherosclerotic plaque regions of METTL14$^{+/-}$/APOE$^{-/-}$ and APOE$^{-/-}$ mice (n=10 per group). All representative images are from mice fed WD. Data are presented as mean ± SEM. Two-tailed unpaired Student's *t*-test was applied to compare the indicated two groups. ***P<0.001.

Supplement Table 1: Full-length sequence of FOXO1 mRNA and GGACT site regions.

5’-1 gatcccgtaa gtcgggcggc ctggtagtcg cagcagccgc tgccgcagcc gccacattca

61 acaggcagca gcgcagcggg cgcgccgctg ggagagcaa gcggcccgcg gcgtccgtcc

121 gtccttccgt ccgcggccct gtcagctgga gcgcggcgca ggctctgccc cggcccggcg

181 gctctggccg gccgtccagt ccgtgcggcg gaccccgagg agcctcgatg tggatggccc

241 cgcgaagtta agttctgggc tcgcgcttcc actccgccgc gccttcctcc cagtttccgt

301 ccgctcgccg caccggcttc gttcccccaa atctcggacc gtcccttcgc gccccctccc

361 cgtccgcccc cagtgctgcg ttctccccct cttggctctc ctgcggctgg gggagggggcg

421 ggggtcacca tggccgaggc gcctcaggtg gtggagatcg acccggactt cgagccgctg

481 ccccggccgc gctcgtgcac ctggccgctg cccaggccgg agtttagcca gtccaactcg

541 gccacctcca gcccggcgcc gtcgggcagc gcggctgcca accccgacgc cgcggcgggc

601 ctgccctcgg cctcggctgc cgctgtcagc gccgacttca tgagcaacct gagcttgctg

661 gaggagagcg aggacttccc gcaggcgccc ggctccgtgg cggcggcggt ggcggcggcg

721 gccgccgcgg ccgccaccgg ggggctgtgc ggggacttcc agggcccgga ggcgggctgc

781 ctgcacccag cgccaccgca gcccccgccg cccgggccgc tgtcgcagca cccgccggtg

841 ccccccgccg ccgctgggcc gctcgcgggg cagccgcgca agagcagctc gtcccgccgc

901 aacgcgtggg gcaacctgtc ctacgccgac ctcatcacca aggccatcga gagctcggcg

961 gagaagcggc tcacgctgtc gcagatctac gagtggatgg tcaagagcgt gccctacttc

1021 aaggataagg gtgacagcaa cagctcggcg ggctggaaga attcaattcg tcataatctg

1081 tccctacaca gcaagttcat tcgtgtgcag aatgaaggaa ctggaaaaag ttcttggtgg

1141 atgctcaatc cagagggtgg caagagcggg aaatctccta ggagaagagc tgcatccatg

1201 gacaacaaca gtaaatttgc taagagccga agccgagctg ccaagaagaa agcatctctc

1261 cagtctggcc aggagggtgc tggggacagc cctggatcac agttttccaa atggcctgca

1321 agccctggct ctcacagcaa tgatgacttt gataactgga gtacatttcg ccctcgaact

1381 agctcaaatg ctagtactat tagtgggaga ctctcaccca ttatgaccga acaggatgat

1441 cttggagaag gggatgtgca ttctatggtg tacccgccat ctgccgcaaa gatggcctct

1501 actttaccca gtctgtctga gataagcaat cccgaaaaca tggaaaatct tttggataat

1561 ctcaaccttc tctcatcacc aacatcatta actgtttcga cccagtcctc acctggcacc

1621 atgatgcagc agacgccgtg ctactcgttt gcgccaccaa acaccagttt gaattcaccc

1681 agcccaaact accaaaaata tacatatggc caatccagca tgagcccttt gccccagatg

1741 cctatacaaa cacttcagga caataagtcg agttatggag gtatgagtca gtataactgt

1801 gcgcctggac tcttgaagga gttgctgact tctgactctc ctccccataa tgacattatg

1861 acaccagttg atcctggggt agcccagccc aacagccggg ttctgggcca gaacgtcatg

1921 atgggcccta attcggtcat gtcaacctat ggcagccagg catctcataa caaaatgatg

1981 aatcccagct cccataccca ccctggacat gctcagcaga catctgcagt taacgggcgt

2041 cccctgcccc acacggtaag caccatgccc cacacctcgg gtatgaaccg cctgacccaa

2101 gtgaagacac ctgtacaagt gcctctgccc caccccatgc agatgagtgc cctgggggc

2161 tactcctccg tgagcagctg caatggctat ggcagaatgg gccttctcca ccaggagaag

2221 ctcccaagtg acttggatgg catgttcatt gagcgcttag actgtgacat ggaatccatc

2281 attcggaatg acctcatgga tggagataca ttggatttta actttgacaa tgtgttgccc

2341 aaccaaagct tcccacacag tgtcaagaca acgacacata gctgggtgtc aggctgaggg

2401 ttagtgagca ggttacactt aaaagtactt cagattgtct gacagcagga actgagagaa

2461 gcagtccaaa gatgtctttc accaactccc ttttagtttt cttggttaaa aaaaaaaaca

2521 aaaaaaaaaa ccctcctttt ttcctttcgt cagacttggc agcaaagaca tttttcctgt

2581 acaggatgtt tgcccaatgt gtgcaggtta tgtgctgctg tagataagga ctgtgccatt
2641 ggaaatttca ttacaatgaa gtgccaaact cactacacca tataattgca gaaaagattt
2701 tcagatcctg gtgtgctttc aagttttgta tataagcagt agatacagat tgtatttgtg
2761 tgtgtttttg gtttttctaa atatccaatt ggtccaagga aagtttatac tctttttgta
2821 atactgtgat gggcctcatg tcttgataag ttaaactttt gtttgtacta cctgttttct
2881 gcggaactga cggatcacaa agaactgaat ctccattctg catctccatt gaacagcctt
2941 ggacctgttc acgttgccac agaattcaca tgagaaccaa gtagcctgtt atcaatctgc
3001 taaattaatg gacttgttaa acttttggaa aaaaaaagat taaatgccag ctttgtacag
3061 gtcttttcta tttttttttg tttattttgt tatttgcaaa tttgtacaaa catttaaatg
3121 gttctaattt ccagataaat gatttttgat gttattgttg ggacttaaga acatttttgg
3181 aatagatatt gaactgtaat aatgttttct taaaactaga gtctactttg ttacatagtc
3241 agcttgtaaa ttttgtggaa ccacaggtat ttggggcagc attcataatt ttcattttgt
3301 attctaactg gattagtact aattttatac atgcttaact ggtttgtaca ctttgggatg
3361 ctacttagtg atgtttctga ctaatcttaa atcattgtaa ttagtacttg catattcaac
3421 gtttcaggcc ctggttgggc aggaaagtga tgtatagtta tggacacttt gcgtttctta
3481 tttaggataa cttaatatgt ttttatgtat gtattttaaa gaaatttcat ctgcttctac
3541 tgaactatgc gtactgcata gcatcaagtc ttctctagag acctctgtag tcctgggagg
3601 cctcataatg tttgtagatc agaaaaggga gatctgcatc taaagcaatg gtcctttgtc
3661 aaacgaggga ttttgatcca cttcaccatt ttgagttgag ctttagcaaa agtttcccct
3721 cataattctt tgctcttgtt tcagtccagg tggaggttgg ttttgtagtt ctgccttgag
3781 gaattatgtc aacactcata cttcatctca ttctcccttc tgccctgcag attagattac
3841 ttagcacact gtggaagttt aagtggaagg agggaattta aaaatgggac ttgagtggtt
3901 tgtagaattt gtgttcataa gttcagatgg gtagcaaatg gaatagaact tacttaaaaa
3961 ttggggagat ttatttgaaa accagctgta agttgtgcat tgagattatg ttaaaagcct
4021 tggcttaaga atttgaaaat ttctttagcc tgtagcaacc taaactgtaa ttcctatcat
4081 tatgttttat tactttccaa ttacctgtaa ctgacagacc aaattaattg gctttgtgtc
4141 ctatttagtc catcagtatt ttcaagtcat gtggaaagcc caaagtcatc acaatgaaga
4201 gaacaggtgc acagcactgt tcctcttgtg ttcttgagaa ggatctaatt tttctgtata
4261 tagcccacat cacacttgct ttgtcttgta tgttaattgc atcttcattg gcttggtatt
4321 tcctaaatgt ttaacaagaa cacaagtgtt cctgataaga tttcctacag taagccagct
4381 ctattgtaag cttcccactg tgatgatcat tttttgaag attcattgaa cagccaccac
4441 tctatcatcc tcattttggg gcagtccaag acatagctgg ttttagaaac ccaagttcct
4501 ctaagcacag cctcccgggt atgtaactga acttggtgcc aaagtacttg tgtactaatt
4561 tctattacta cgtactgtca ctttcctccc gtgccattac tgcatcataa tacaaggaac
4621 ctcagagccc ccatttgttc attaaagagg caactacagc caaaatcact gttaaaatct
4681 tactacttca tggagtagct cttaggaaaa tatatcttcc tcctgagtct gggtaattat
4741 acctctccca agcccccatt gtgtgttgaa atcctgtcat gaatccttgg tagctctctg
4801 agaacagtga agtccaggga aaggcatctg gtctgtctgg aaagcaaaca ttatgtggcc
4861 tctggtagtt tttttcctgt aagaatactg actttctgga gtaatgagta tatatcagtt
4921 attgtacatg attgctttgt gaaatgtgca aatgatatca cctatgcagc cttgtttgat
4981 ttattttctc tggtttgtac tgttattaaa agcatattgt attatagagc tattcagata
5041 ttttaaatat aaagatgtat tgtttccgta atatagacgt atggaatata tttaggtaat
5101 agatgtatta cttggaaagt tctgctttga caaactgaca aagtctaaat gagcacatgt
5161 atcccagtga gcagtaaatc aatggaacat cccaagaaga ggataaggat gcttaaaatg

5221 gaaatcattc tccaacgata tacaaattgg acttgttcaa ctgctggata tatgctacca
5281 ataaccccag ccccaactta aaattcttac attcaagctc ctaagagttc ttaatttata
5341 actaatttta aaagagaagt ttcttttctg gttttagttt gggaataatc attcattaaa
5401 aaaaatgtat tgtggtttat gcgaacagac caacctggca ttacagttgg cctctccttg
5461 aggtgggcac agcctggcag tgtggccagg ggtggccatg taagtcccat caggacgtag
5521 tcatgcctcc tgcatttcgc tacccgagtt tagtaacagt gcagattcca cgttcttgtt
5581 ccgatactct gagaagtgcc tgatgttgat gtacttacag acacaagaac aatctttgct
5641 ataattgtat aaagccataa atgtacataa attatgttta aatggcttgg tgtctttctt
5701 ttctaattat gcagaataag ctctttatta ggaatttttt gtgaagctat taaatacttg
5761 agttaagtct tgtcagcca-3’

The red font indicates the CDS area, and the yellow background font indicates the GGACT area.

Table S2: Weight gain and plasma lipid profiles between $APOE^{-/-}$ and $METTL14^{+/-}/APOE^{-/-}$ mice.

	$APOE^{-/-}$	$METTL14^{+/-}/APOE^{-/-}$	*P* Value
Body Weight (g)	23.71±1.88	23.21±1.54	0.527
LDL (mg/dL)	355.73±17.74	343.84±13.81	0.113
HDL (mg/dL)	83.61±6.55	85.60±5.68	0.475
TG (mg/dL)	138.80±6.09	133.90±7.32	0.122

Table S2. $APOE^{-/-}$ and $METTL14^{+/-}/APOE^{-/-}$ mice were fed with WD for 12 weeks and the body weight (n=12-15 for each group) and plasma lipid profiles (LDL, HDL, and TG) was measured by Elisa (n=10). All data are mean ± SEM. Significance was determined using a 2-tailed unpaired Student *t* test.
